# *N6*-Methyladenosine in *Flaviviridae* Viral RNA Genomes Regulates Infection

**DOI:** 10.1016/j.chom.2016.09.015

**Published:** 2016-11-09

**Authors:** Nandan S. Gokhale, Alexa B.R. McIntyre, Michael J. McFadden, Allison E. Roder, Edward M. Kennedy, Jorge A. Gandara, Sharon E. Hopcraft, Kendra M. Quicke, Christine Vazquez, Jason Willer, Olga R. Ilkayeva, Brittany A. Law, Christopher L. Holley, Mariano A. Garcia-Blanco, Matthew J. Evans, Mehul S. Suthar, Shelton S. Bradrick, Christopher E. Mason, Stacy M. Horner

**Affiliations:** 1 Department of Molecular Genetics and Microbiology, Duke University Medical Center, Durham, NC 27710, USA; 2 Department of Medicine, Duke University Medical Center, Durham, NC 27710, USA; 3 Department of Physiology and Biophysics, Weill Cornell Medicine, New York, NY 10021, USA; 4 Department of Microbiology, Icahn School of Medicine at Mount Sinai, New York, NY 10029, USA; 5 Division of Infectious Diseases, Department of Pediatrics, Emory University School of Medicine, Atlanta, GA 30322, USA; 6 Emory Vaccine Center, Yerkes National Primate Research Center, Atlanta, GA 30329, USA; 7 Duke Molecular Physiology Institute, Duke University, Durham NC 27701, USA; 8 Department of Biochemistry and Molecular Biology, University of Texas Medical Branch, Galveston, TX 77555, USA; 9 The HRH Prince Alwaleed Bin Talal Bin Abdulaziz Alsaud Institute for Computational Biomedicine, Weill Cornell Medicine, New York, NY 10021, USA; 10 The Feil Family Brain and Mind Research Institute, Weill Cornell Medicine, New York, NY 10021, USA; 11 Programme in Emerging Infectious Disease, Duke-NUS Medical School, Singapore 169857, Singapore; 12 Tri-Institutional Program in Computational Biology and Medicine, New York City, NY 10065, USA

**Keywords:** RNA-modifications, m^6^A, N6-methyladenosine, HCV, Flaviviridae, viral particle production, Zika, dengue, West Nile, yellow fever

## Abstract

The RNA modification *N6*-methyladenosine (m^6^A) post-transcriptionally regulates RNA function. The cellular machinery that controls m^6^A includes methyltransferases and demethylases that add or remove this modification, as well as m^6^A-binding YTHDF proteins that promote the translation or degradation of m^6^A-modified mRNA. We demonstrate that m^6^A modulates infection by hepatitis C virus (HCV). Depletion of m^6^A methyltransferases or an m^6^A demethylase, respectively, increases or decreases infectious HCV particle production. During HCV infection, YTHDF proteins relocalize to lipid droplets, sites of viral assembly, and their depletion increases infectious viral particles. We further mapped m^6^A sites across the HCV genome and determined that inactivating m^6^A in one viral genomic region increases viral titer without affecting RNA replication. Additional mapping of m^6^A on the RNA genomes of other *Flaviviridae*, including dengue, Zika, yellow fever, and West Nile virus, identifies conserved regions modified by m^6^A. Altogether, this work identifies m^6^A as a conserved regulatory mark across *Flaviviridae* genomes.

## Highlights

• The RNA genomes of HCV, ZIKV, DENV, YFV, and WNV contain m^6^A modification
• The cellular m^6^A machinery regulates HCV infectious particle production
• YTHDF proteins reduce HCV particle production and localize at viral assembly sites
• m^6^A-abrogating mutations in HCV E1 increase infectious particle production

## Graphical Abstract

**Figure:**
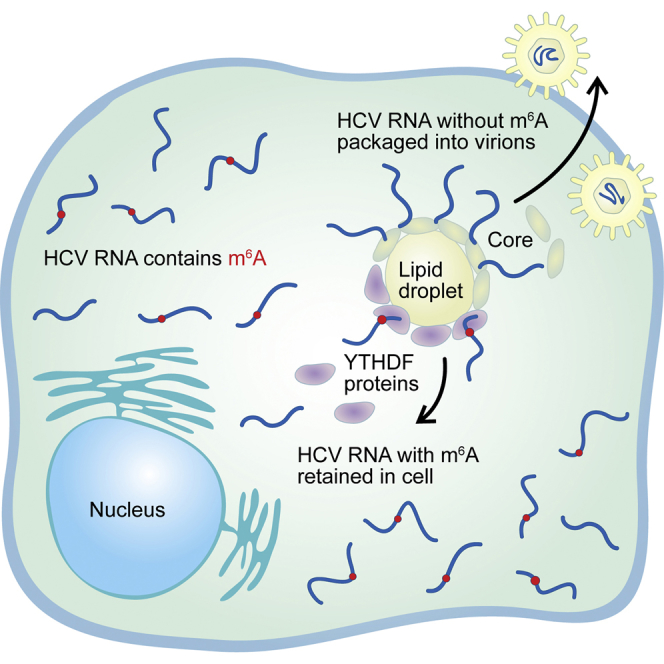

## Teaser

*N6*-methyladenosine (m^6^A) post-transcriptionally regulates RNA function. Gokhale et al. demonstrate that the RNA genomes of HCV, ZIKV, DENV, YFV, and WNV are modified by m^6^A. Depletion of cellular machinery that regulates m^6^A or introduction of m^6^A-abrogating mutations within HCV RNA increase viral particle production, suggesting that m^6^A negatively regulates HCV.

## Introduction

The chemical modification of RNA is an important post-transcriptional regulator of RNA. Of the many known RNA modifications, *N6*-methyladenosine (m^6^A) is the most abundant internal modification of eukaryotic mRNAs, contributing to RNA structure, localization, and function (Fu et al., 2014, Meyer and Jaffrey, 2014). m^6^A regulates many biological processes, including stress responses, fertility, stem cell differentiation, circadian rhythms, microRNA (miRNA) biogenesis, and cancer (Li and Mason, 2014, Saletore et al., 2012, Yue et al., 2015, Zhou et al., 2015). However, little is known about its effects on microbial infection. m^6^A has long been known to be present in the RNA transcripts of viruses with nuclear replication, such as influenza A virus, simian virus 40, Rous sarcoma virus, avian sarcoma virus, and adenovirus (Dimock and Stoltzfus, 1977, Kane and Beemon, 1985, Krug et al., 1976, Lavi and Shatkin, 1975, Sommer et al., 1976). More recently, we and others have shown that m^6^A serves as a positive regulator of HIV-1, a retrovirus with a nuclear replication step (Kennedy et al., 2016, Lichinchi et al., 2016, Tirumuru et al., 2016). However, a role for m^6^A in regulating the life cycle of viruses that replicate exclusively in the cytoplasm, such as viruses within the *Flaviviridae* family, has been unexplored. *Flaviviridae*, including Zika virus (ZIKV), dengue virus (DENV), West Nile virus (WNV), yellow fever virus (YFV), and hepatitis C virus (HCV), represent both established and emerging pathogens. They contain a positive-sense, single-stranded RNA genome that encodes a viral polyprotein and use similar replication strategies. RNA-based regulation of these viral genomes plays a fundamental role in their infection, such as the liver-specific miRNA miR-122 for HCV replication, RNA structural elements for HCV and DENV replication, and 2′-O methylation of the 5′ cap of WNV RNA for immune evasion and WNV replication (Bidet and Garcia-Blanco, 2014, Hyde et al., 2014, Jopling et al., 2005, Mauger et al., 2015, Pirakitikulr et al., 2016).

The cellular machinery that regulates m^6^A includes proteins that act as writers, erasers, and readers of m^6^A. The addition of m^6^A on mRNA, which occurs at the consensus motif DRA^m^CH (where D = G/A/U, R = G > A, and H = U/C/A), is mediated by a methyltransferase complex containing the methyltransferase-like (METTL) enzymes METTL3 and METTL14 and the cofactors Wilms tumor 1-associated protein (WTAP) and KIAA1429 (Fu et al., 2014, Liu et al., 2014, Meyer and Jaffrey, 2014, Schwartz et al., 2014, Yue et al., 2015). The removal of m^6^A from mRNA is catalyzed by the demethylases fat mass and obesity-associated protein (FTO) or α-ketoglutarate-dependent dioxygenase AlkB homolog 5 (ALKBH5) (Jia et al., 2011, Zheng et al., 2013). The cytoplasmic YTH-domain family 1 (YTHDF1), YTHDF2, and YTHDF3 proteins bind to m^6^A through their C-terminal YTH domain. Functionally, YTHDF1 promotes the translation of m^6^A-modified mRNA, while YTHDF2 targets m^6^A-modified mRNAs for degradation (Wang et al., 2014, Wang et al., 2015). The function of YTHDF3 is still unknown. The discovery of these proteins and the development of high-throughput m^6^A-mapping techniques have led to many insights into the function of m^6^A (Dominissini et al., 2012, Fu et al., 2014, Linder et al., 2015, Meyer et al., 2012). Nonetheless, many aspects of the regulation of specific mRNAs by m^6^A remain unexplored.

Here, we define a role for m^6^A in regulating the life cycle of HCV. We demonstrate that the m^6^A methyltransferases negatively regulate the production of infectious HCV particles and that the m^6^A-binding YTHDF proteins all relocalize to sites of HCV particle production and suppress this stage of viral infection. We map m^6^A across the HCV RNA genome and show that preventing m^6^A at one of these regions enhances viral titer by increasing the interaction of the HCV RNA with the HCV Core protein. Finally, we describe viral RNA m^6^A-epitranscriptomic maps for several other *Flaviviridae*, including ZIKV, DENV, WNV, and YFV. Altogether, our data reveal that m^6^A regulates HCV infection and set the stage for the exploration of the function of m^6^A within the broader *Flaviviridae* family of viruses.

## Results

### The m^6^A Machinery Regulates HCV Particle Production

**Figure 1:**
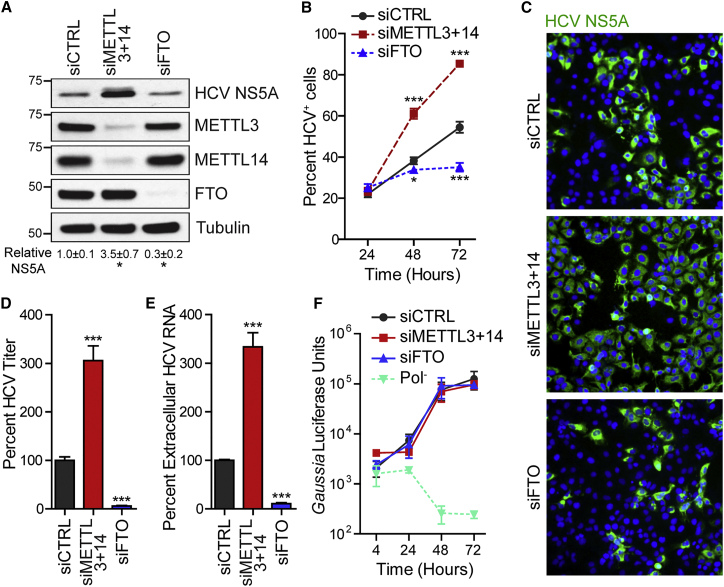
The m^6^A Machinery Regulates Infectious HCV Particle Production (A) Immunoblot analysis of extracts of HCV-infected Huh7 cells (72 hpi) treated with siRNAs. NS5A levels were quantified relative to tubulin (n = 3). ^∗^p ≤ 0.05 by unpaired Student’s t test. (B) Percentage of HCV+ cells by immunostaining of NS5A and nuclei (DAPI) after siRNA. n = 3, with ≥5,000 cells counted per condition. ^∗^p ≤ 0.05, ^∗∗∗^p ≤ 0.001 by two-way ANOVA with Bonferroni correction. (C) Representative fields of HCV-infected cells (NS5A^+^, green) and nuclei (DAPI, blue) at 72 hpi from (B). (D and E) FFA of supernatants harvested from Huh7 cells 72 hpi after siRNA treatment (D). HCV RNA in supernatants harvested from Huh7 cells 72 hpi after siRNA treatment as quantified by qRT-PCR (E). Data are presented as the percentage of viral titer or RNA relative to control siRNA. ^∗∗∗^p ≤ 0.001 by unpaired Student’s t test. Values are the mean ± SEM of three experiments in triplicate. (F) *Gaussia* luciferase assay to measure HCV luciferase reporter (JFH1-QL/GLuc2A) transfected in Huh7.5 CD81 KO cells after siRNA treatment. Pol^−^, lethal mutation in HCV NS5B polymerase. Values in (B) and (F) represent the mean ± SD (n = 3) and are representative of three independent experiments. See also Figure S1.

To determine whether m^6^A regulates HCV infection, we depleted the m^6^A methyltransferases METTL3 and METTL14 (METTL3+14) by small interfering RNA (siRNA) in Huh7 liver hepatoma cells and infected these cells with HCV. Immunoblot analysis of cell extracts harvested at 72 hr post-infection (hpi) revealed that METTL3+14 depletion significantly increased the abundance of the HCV NS5A protein, a marker of viral replication, relative to its level in cells treated with non-targeting control siRNA (Figure 1A). Conversely, depletion of the m^6^A demethylase FTO decreased HCV NS5A levels relative to the control (Figure 1A). Furthermore, we found that the percentage of HCV-positive cells increased after METTL3+14 depletion and decreased after FTO depletion (Figures 1B, 1C, and S1A). This change in HCV-positive cells occurred only after 24 hpi, suggesting that viral entry was unaffected by m^6^A machinery depletion. Depletion of the m^6^A machinery did not impair cell viability during infection (Figure S1B). In addition, HCV infection slightly reduced METTL3 protein levels in total cellular extracts, while METTL14 and FTO were unaffected (Figures S1C and S1D). Thus, the m^6^A methyltransferases negatively regulate HCV infection, while the m^6^A demethylase positively regulates HCV infection.

We next defined the stage of the HCV life cycle regulated by the m^6^A machinery. Depletion of METTL3+14 significantly increased the production of infectious virus and viral RNA in the supernatant compared to control siRNA at 72 hpi (Figures 1D and 1E). Conversely, depletion of FTO decreased infectious virus and HCV RNA in the supernatant (Figures 1D and 1E) without altering the viral-specific infectivity (Figure S1E). Depletion of ALKBH5 did not affect viral titer or protein levels, indicating that this demethylase does not influence the HCV life cycle (Figure S1F). We next tested whether the altered HCV titer after m^6^A machinery depletion was due to altered viral RNA replication. In these experiments, we used Huh7.5 CD81 knockout (KO) cells, in which essential HCV entry factor CD81 (Zhang et al., 2004) was deleted by clustered regularly interspaced short palindromic repeats/Cas9 (CRISPR/Cas9), resulting in cells permissive for HCV RNA replication and viral particle production following viral RNA transfection that are unable to support subsequent rounds of viral infection (Figures S1G–S1I). In these cells, we depleted METTL3+14 or FTO by siRNA, transfected the cells with in vitro transcribed RNA of the HCV reporter virus JFH1-QL/GLuc2A, and measured HCV RNA replication by assaying for secreted *Gaussia* luciferase (Yamane et al., 2014). Depletion of METTL3+14 or FTO had no effect on *Gaussia* luciferase levels compared to control over the time course, while our negative control RNA containing a point mutation in the viral RNA-dependent RNA polymerase (Pol^−^) did not replicate (Figure 1F). These data indicate that m^6^A dynamics do not regulate HCV translation or RNA replication but do regulate the production or release of infectious viral particles.

Changes in expression of the m^6^A machinery have been shown to affect cellular gene expression (Dominissini et al., 2012, Meyer et al., 2012, Wang et al., 2014), which could indirectly regulate the HCV life cycle, for example, by inducing antiviral interferon-stimulated genes (ISGs). While we did not find consistent changes in ISG mRNA levels following loss of the m^6^A machinery during HCV infection (48 hpi), FTO depletion slightly increased the expression of *IFITM1*, which is known to restrict HCV entry (Figure S1J) (Wilkins et al., 2013). This slight increase occurred at both 24 and 48 hpi, although the percentage of HCV-positive cells following FTO depletion is the same as control and METTL3+14 depletion at 24 hpi (Figures 1B, S1J, and S1K). Therefore, the observed changes in infectious virus following depletion of the m^6^A machinery are not solely a result of an altered antiviral response in these cells. Rather, these data suggest that m^6^A acts directly on the HCV RNA genome to regulate HCV particle production.

### The m^6^A-Binding YTHDF Proteins Negatively Regulate HCV Particle Production

**Figure 2:**
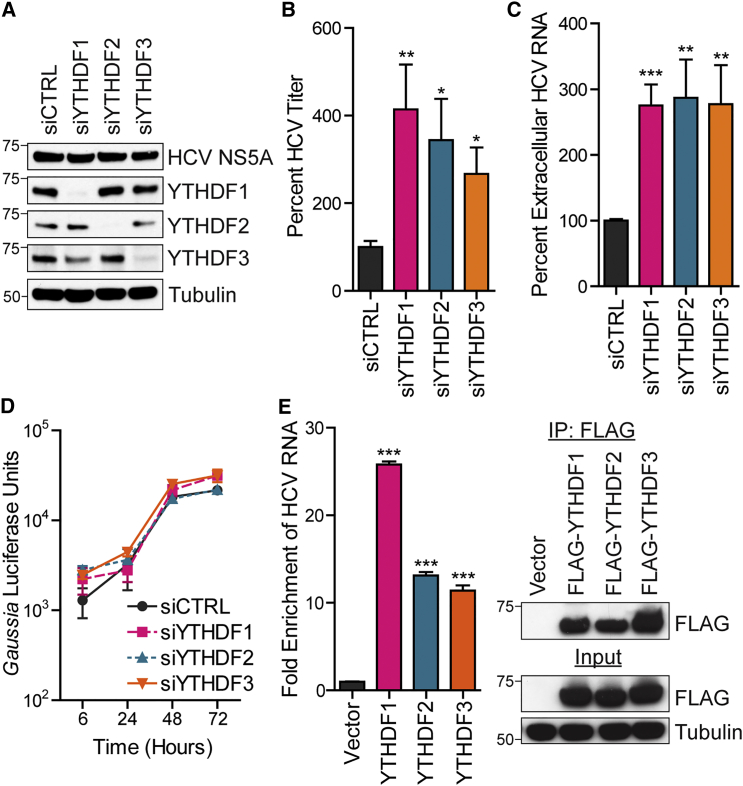
The m^6^A-Binding YTHDF Proteins Negatively Regulate Infectious HCV Particle Production (A) Immunoblot analysis of extracts of HCV-infected Huh7 cells (48 hpi) treated with indicated siRNAs. (B and C) FFA of supernatants harvested from Huh7 cells at 72 hpi after siRNA treatment (B). HCV RNA in supernatants harvested from Huh7 cells 72 hpi after siRNA treatment was quantified by qRT-PCR (C). Data were analyzed as the percentage of titer or HCV RNA relative to cells treated with control siRNA. Values represent the mean ± SEM of three (C) or four (B) experiments done in triplicate. (D) *Gaussia* luciferase assay to measure HCV luciferase reporter (JFH1-QL/GLuc2A) transfected in Huh7.5 CD81 KO cells after siRNA. (E) Enrichment of HCV RNA following immunoprecipitation of FLAG-tagged YTHDF from extracts of Huh7 cells after 48 hpi. Left: captured HCV RNA was quantified by qRT-PCR as the percentage of input and graphed as fold enrichment relative to vector. Right: immunoblot analysis of immunoprecipitated extracts and input. For (D) and (E), data are representative of three experiments and show the mean ± SD (n = 3). ^∗^p ≤ 0.05, ^∗∗^p ≤ 0.01, ^∗∗∗^p ≤ 0.001 by unpaired Student’s t test. See also Figure S2.

Given that the m^6^A machinery regulates infectious HCV particle production, we next tested whether the known mediators of m^6^A function, the RNA-binding YTHDF proteins, similarly regulate the HCV life cycle. Depletion of any of the YTHDF proteins did not increase HCV NS5A protein levels at 48 hpi or HCV RNA replication of the HCV reporter (JFH1-QL/GLuc2A) over 72 hr in Huh7.5 CD81 KO cells. However, by 72 hpi, the levels of infectious HCV particles and HCV RNA in the supernatant were increased at least 2-fold compared to control (Figures 2A–2D). Depletion of YTHDF proteins did not affect cell viability, and HCV infection did not alter their expression (Figures S2A and S2B). Collectively, these data suggest that the YTHDF proteins negatively regulate infectious HCV production without affecting overall HCV RNA replication.

We next tested whether YTHDF proteins bind to HCV RNA by RNA immunoprecipitation (RIP). We found that FLAG-YTHDF ribonucleoprotein (RNP) complexes enriched HCV RNA relative to the input, demonstrating that these proteins bind to viral RNA (Figure 2E). Thus, YTHDF protein binding to HCV RNA may mediate regulation of HCV particle production. This led us to examine the subcellular localization of the YTHDF proteins during HCV infection.

### YTHDF Proteins Relocalize to Lipid Droplets during HCV Infection

**Figure 3:**
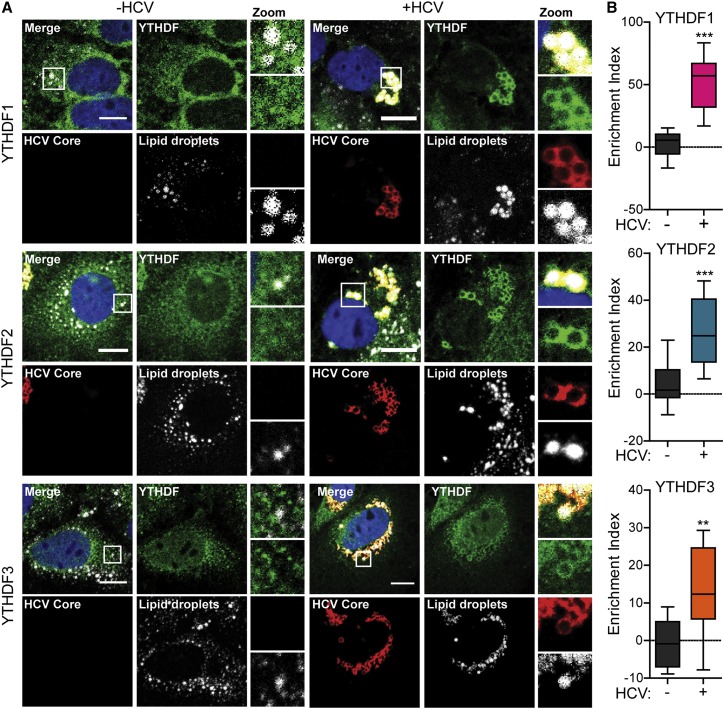
YTHDF Proteins Relocalize to Lipid Droplets during HCV Infection (A) Confocal micrographs of HCV-infected or uninfected Huh7 cells (48 hpi) immunostained with antibodies to YTHDF (green) and HCV Core (red) proteins. Lipid droplets (gray) and nuclei (blue) were labeled with BODIPY and DAPI, respectively. Zoom panels are derived from the white box in the merge panels. Scale bar, 10 μm. (B) Enrichment of YTHDF proteins around lipid droplets was quantified using ImageJ from more than ten cells analyzed and graphed in box-and-whisker plots, representing the minimum, first quartile, median, third quartile, and maximum. ^∗∗^p ≤ 0.01, ^∗∗∗^p ≤ 0.001 by unpaired Student’s t test. See also Figure S3.

HCV particle assembly occurs around cytosolic lipid droplets in close association with endoplasmic reticulum (ER) membranes. HCV RNA and proteins, including NS5A and Core (the capsid protein), as well as several host RNA-binding proteins that regulate HCV infection, accumulate around lipid droplets (Ariumi et al., 2011, Chatel-Chaix et al., 2013, Miyanari et al., 2007, Pager et al., 2013, Poenisch et al., 2015). Therefore, we analyzed the subcellular localization of YTHDF proteins after HCV infection in Huh7 cells by confocal microscopy. While YTHDF proteins were distributed in the cytoplasm in uninfected cells, in HCV-infected cells all three YTHDF proteins (both endogenous and overexpressed) were enriched around lipid droplets (Figures 3 and S3A), in which they colocalized with the HCV Core protein. We did not observe this relocalization in Huh7 cells stably expressing a subgenomic HCV replicon that lacks the HCV structural genes and cannot produce viral particles (Figure S3B) (Wang et al., 2003), suggesting that a fully productive HCV infection is required to trigger the relocalization of the YTHDF proteins around lipid droplets.

### HCV RNA Is Modified by m^6^A

**Figure 4:**
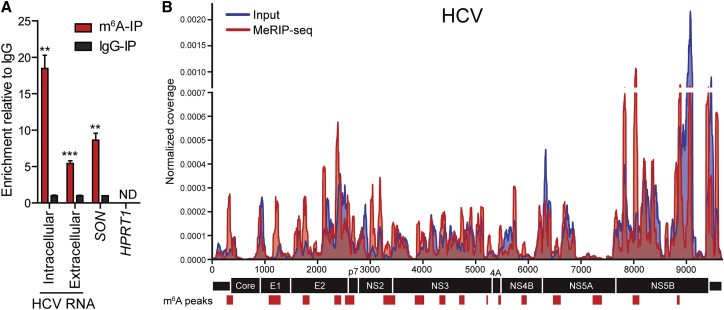
HCV RNA Is Modified by m^6^A (A) MeRIP-qRT-PCR analysis of intracellular or supernatant RNA harvested from HCV-infected Huh7.5 cells (72 hpi) and immunoprecipitated with anti-m^6^A or IgG. Eluted RNA is quantified as a percentage of input. Values are the mean ± SD (n = 3). ^∗∗^p ≤ 0.01, ^∗∗∗^p ≤ 0.001 by unpaired Student’s t test. (B) Map of m^6^A-binding sites in the HCV RNA genome by MeRIP-seq (representative of two independent samples) of RNA isolated from HCV-infected Huh7 cells. Read coverage, normalized to the total number of reads mapping to the viral genome for each experiment, is in red for MeRIP-seq and in blue for input RNA-seq. Red bars indicate m^6^A peaks identified in duplicate experiments by MeRIPPeR analysis (FDR-corrected q value < 0.05). See also Figures S4 and S6 and Tables S1 and S2.

We and others mapped m^6^A on HIV-1 mRNA and showed that it regulates viral gene expression (Kennedy et al., 2016, Lichinchi et al., 2016, Tirumuru et al., 2016). Although m^6^A has not been found in RNAs from viruses that replicate in the cytoplasm, our findings (Figures 1, 2, and 3) led us to hypothesize that the HCV RNA genome is modified by m^6^A during infection. To test this, we used an antibody that specifically recognizes m^6^A to perform methyl-RNA immunoprecipitation (MeRIP) on total RNA harvested from HCV-infected cells, followed by qRT-PCR to detect enriched RNAs. HCV RNA in the eluate was specifically enriched by the anti-m^6^A antibody, but not immunoglobulin G (IgG), as was known m^6^A-modified mRNA *SON*, but not an mRNA with little m^6^A modification, *HPRT1* (Figure 4A) (Wang et al., 2014). Ultra-high-pressure liquid chromatography-tandem mass spectrometry (UPLC-MS/MS) analysis of viral RNA captured from HCV-infected Huh7 cells using specific antisense oligonucleotides proved that HCV RNA contains m^6^A, with a ratio of m^6^A/A of approximately 0.16% (Figures S4A and S4B). The anti-m^6^A antibody did not enrich HCV RNA isolated from cell supernatants to the same degree as intracellular viral RNA (Figure 4A). We next mapped the sites of the HCV RNA genome modified by m^6^A using MeRIP followed by sequencing (MeRIP-seq), as previously described (Dominissini et al., 2013, Meyer et al., 2012). We identified 19 peaks across the HCV RNA genome common to both experimental replicates (Figures 4B and S6; Table S1). These data present evidence that HCV, which replicates exclusively in the cytoplasm, is marked by m^6^A during infection.

As HCV replicates in the cytoplasm in association with intracellular membranes, for its RNA to undergo m^6^A modification, the m^6^A methyltransferases must also exist in the cytoplasm. Our immunoblot analysis of isolated nuclear and cytoplasmic fractions from mock or HCV-infected Huh7 cells reveals that METTL3, METTL14, and FTO are all present in both the nucleus and the cytoplasm, where they could interact with viral RNA (Figures S1C and S1D). This is in concordance with reports that have detected both METTL3 and m^6^A-methyltransferase activity in cytoplasmic extracts (Chen et al., 2015, Harper et al., 1990, Lin et al., 2016). Therefore, these data reveal that the m^6^A machinery are in the cytoplasm, where they can modify cytoplasmic HCV RNA.

Because the cellular function of m^6^A is carried out by the YTHDF proteins, which are bound to HCV RNA (Figure 2E), we hypothesized that one or more of the YTHDF proteins would bind to functionally relevant m^6^A sites on the HCV RNA genome. We directly mapped these YTHDF-binding sites on the viral genome using photoactivatable ribonucleoside-enhanced crosslinking and immunoprecipitation (PAR-CLIP) in HCV-infected Huh7 single-cell clones stably expressing these proteins or GFP (Figure S4C) (Hafner et al., 2010, Kennedy et al., 2016). We identified 42 different sites on the HCV RNA genome that were bound by at least one YTHDF protein, not by GFP, and contained the characteristic T-to-C transition that derives from reverse transcription of cross-linked 4SU residues (Table S2). Surprisingly, only two high-confidence YTHDF-binding sites (bound by more than one YTHDF protein) overlapped with the m^6^A peaks identified by all replicates of MeRIP-seq, and only 55% of the YTHDF-binding sites contained the DRA^m^CH motif required for m^6^A (Table S2). Altogether, these data build a map of m^6^A- and YTHDF-binding sites on the HCV RNA genome.

### m^6^A-Abrogating Mutations in the HCV E1 Genomic Region Increase Viral Particle Production

**Figure 5:**
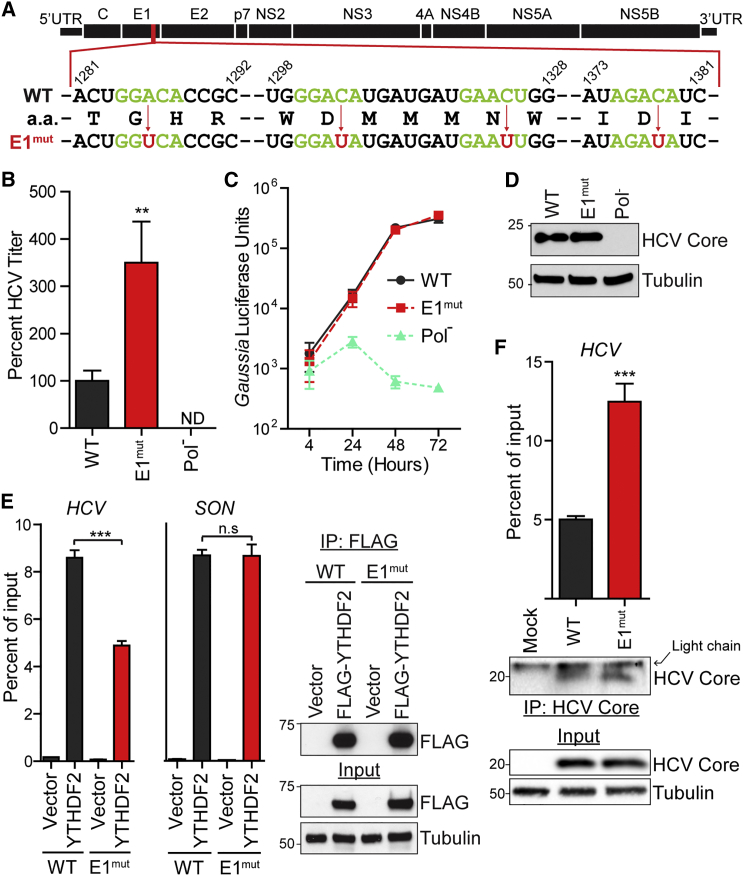
m^6^A-Abrogating Mutations in E1 Increase Infectious HCV Particle Production (A) Schematic of the HCV genome with the mutation scheme for altering A or C residues (red arrows) to make the E1^mut^ virus. Consensus m^6^A motifs (green) and inactivating mutations (red) are shown. Dashes represent nucleotides not shown. Genomic indices match the HCV JFH-1 genome (AB047639). (B) FFA of supernatants harvested from Huh7 cells after electroporation of WT or E1^mut^ HCV RNA (48 hr) and analyzed as the percentage of viral titer relative to WT. (C) *Gaussia* luciferase assay to measure levels of the WT, E1^mut^, or Pol^−^ HCV luciferase reporter (JFH1-QL/GLuc2A) transfected in Huh7.5 CD81 KO cells. (D) Immunoblot analysis of extracts of WT, E1^mut^, or Pol^−^ JFH1-QL/GLuc2A transfected in Huh7.5 CD81 KO cells. (E) Enrichment of WT or E1^mut^ reporter RNA or *SON* mRNA by immunoprecipitation of FLAG-YTHDF2 or vector from extracts of Huh7 cells. Captured RNA was quantified by qRT-PCR and graphed as the percentage of input. Right: immunoblot analysis of anti-FLAG immunoprecipitated extracts and input. (F) Enrichment of WT or E1^mut^ HCV RNA by immunoprecipitation of HCV Core from extracts of Huh7 cells electroporated with the indicated viral genomes (48 hr). Lower: immunoblot analysis of anti-Core immunoprecipitated extracts and input. Data are representative of two (D and E) or three (B, C, and F) experiments and presented as the mean ± SD (n = 3). ^∗^p ≤ 0.05, ^∗∗^p ≤ 0.01, ^∗∗∗^p ≤ 0.001 by unpaired Student’s t test. See also Figure S5.

To elucidate the functional relevance of a specific m^6^A site on the HCV genome, we made mutations in the genome to inactivate this modification. We identified only one region of the HCV genome, within the viral E1 gene, that both contains m^6^A and is bound by YTHDF proteins at sites with consensus DRA^m^CH motifs (Tables S1 and S2). This region of the genome has been shown to lack major RNA secondary structure (Pirakitikulr et al., 2016) and contains a cluster of four potential m^6^A sites (Figure 5A). Comparative sequence analysis of the nucleotides in these sites revealed that the first m^6^A site is identical in 72 strains of genotype 2A, while the m^6^A motif in the latter three sites is conserved among 26 representative strains of HCV from different genotypes (Figure S5A). We then mutated either the A or the C within the consensus site to a U in the four identified m^6^A sites in the E1 gene to construct HCV-E1^mut^. These mutations abrogate the potential for m^6^A modification (Kane and Beemon, 1987) without altering the encoded amino acid sequence (Figure 5A).

To determine the role of these m^6^A sites in the HCV life cycle, we electroporated wild-type (WT) and E1^mut^ HCV RNA into Huh7 cells and measured the production of infectious virus at 48 hpi. E1^mut^ produced nearly 3-fold more viral titer in supernatant than WT, while the Pol^−^ RNA did not produce titer (Figure 5B). E1^mut^ also increased both intracellular and extracellular titer, suggesting that these mutations increased viral particle assembly (Figure S5B). To determine whether abrogation of the E1 m^6^A sites affected HCV RNA replication, we then measured replication of the WT or E1^mut^ JFH1-QL/GLuc2A reporter after transfection into Huh7.5 CD81 KO cells. The E1 mutations did not alter HCV RNA replication over a time course (Figure 5C) or the levels of viral Core protein (Figure 5D). Altogether, these data suggest that m^6^A within the E1 gene negatively regulates infectious HCV particle production, similar to our findings with depletion of the m^6^A methyltransferases and YTHDF proteins.

While the YTHDF proteins bind to multiple sites on HCV RNA, comparison of MeRIP-seq with the PAR-CLIP data suggests that their binding to the HCV RNA genome is not always m^6^A dependent (Figure 4B; Table S2). Therefore, to test whether the m^6^A-abrogating mutations in E1 affect binding by YTHDF proteins within this region, we measured FLAG-YTHDF2 binding to a reporter RNA containing 100 nucleotides of WT or E1^mut^, allowing us to isolate the interaction of a single m^6^A region with a single YTHDF protein. Mutation of the m^6^A sites within the E1 region reduced binding of FLAG-YTHDF2 by 50% compared to the WT by YTHDF2 RIP, while FLAG-YTHDF2 bound equally to known m^6^A-modified mRNA *SON* in both conditions (Figure 5E). Furthermore, depletion of YTHDF1 did not increase extracellular HCV RNA produced by cells infected with E1^mut^ HCV over cells treated with control siRNA (Figure S5C).

The HCV Core protein binds to the HCV RNA genome during assembly of viral particles. Core protein is known to bind to HCV RNA around the E1 region that contains our identified m^6^A sites (Shimoike et al., 1999). To test whether m^6^A in E1 influences Core binding to viral RNA, we immunoprecipitated Core RNP complexes from cells electroporated with WT or E1^mut^ HCV RNA. We found that mutation of the m^6^A sites within the E1 region increases HCV RNA binding to the Core protein by nearly 2-fold compared to WT (Figure 5F). Altogether, these data suggest that YTHDF proteins bind to the m^6^A sites within the HCV E1 region to mediate the negative regulation of infectious HCV particle production, while the Core protein binds to viral RNA genomes lacking m^6^A within the E1 region for packaging into nascent viral particles.

### Mapping of m^6^A within the Viral RNA Genomes of the *Flaviviridae* Family of Viruses

**Figure 6:**
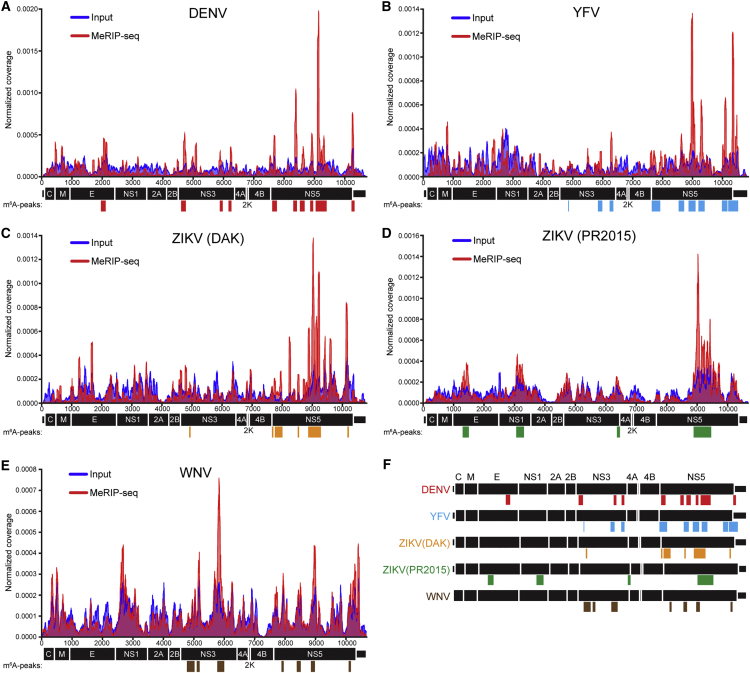
Mapping m^6^A in the RNA Genomes of *Flaviviridae* (A–E) Read coverage of *Flaviviridae* genomes of (A) DENV, (B) YFV, (C) ZIKV (DAK), (D) ZIKV (PR2015), and (E) WNV for one replicate of MeRIP-seq (red), and input RNA-seq (blue) from matched samples. Colored bars indicate m^6^A peaks identified by MeRIPPeR analysis. (n = 2; FDR-corrected q value < 0.05). (F) Alignment of replicate m^6^A sites in the genomes of DENV (red), YFV (blue), ZIKV (DAK) (orange), ZIKV (PR2015) (green), and WNV (brown). See also Figure S6 and Table S3.

Because we found that the HCV RNA genome contains m^6^A, we wanted to investigate the location of m^6^A on the RNA genomes of other members of the *Flaviviridae* family. We performed MeRIP-seq in duplicate on RNA isolated from Huh7 cells infected with DENV (DENV2-NGC), YFV (17D), WNV (TX), and ZIKV (PR2015 or DAK). Our data identified reproducible m^6^A sites within all five viral genomes (Figures 6A–6E and S6; Table S3). Some m^6^A sites on these viral genomes occurred within similar genetic regions among all *Flaviviridae* (Figure 6F). In particular, the NS3 and NS5 genes contained m^6^A peaks, reminiscent of the pattern on the HCV RNA genome and suggesting a conserved role for these sites in regulating these viral life cycles. Furthermore, similar to HCV, DENV and ZIKV (PR2015) contained an m^6^A peak in the envelope gene. Therefore, these data suggest a potentially conserved set of m^6^A-epitranscriptome sites in the *Flaviviridae* family that could regulate viral RNA function, virulence, and transmission.

## Discussion

The function of m^6^A in regulating host and viral infection is only now emerging, even though nuclear-replicating viruses have been known to contain m^6^A since the 1970s (Dimock and Stoltzfus, 1977, Kane and Beemon, 1985, Krug et al., 1976, Lavi and Shatkin, 1975, Sommer et al., 1976). Recent studies have established a pro-viral role for m^6^A during HIV-1 infection (Kennedy et al., 2016, Lichinchi et al., 2016, Tirumuru et al., 2016). In our study, in which we define function for m^6^A and its cellular machinery in regulating the positive-strand RNA genome of the cytoplasmic virus HCV, we find that m^6^A negatively regulates HCV particle production. Furthermore, we find that the positive-strand RNA genomes of other viruses within the *Flaviviridae* family, including two strains of ZIKV, are modified by m^6^A in conserved genomic regions. Altogether, this work reveals that *Flaviviridae* RNA genomes harbor RNA regulatory marks that could affect their life cycles and virulence.

The known enzymes and RNA-binding proteins that regulate m^6^A also regulate the life cycle of HCV. Depletion of the m^6^A methyltransferases METTL3 and METTL14 increases the rate of HCV infection by promoting infectious viral particle production without affecting viral RNA replication. Depletion of the m^6^A demethylase FTO, but not ALKBH5, has the opposite effect (Figure 1). These effects do not appear to be caused by dysregulated induction of host ISGs after depletion of the m^6^A machinery, because changes in ISG expression were minimal (Figure S1). Instead, we hypothesize that the m^6^A machinery directly modulates the levels of m^6^A on the HCV genome to regulate its function, and this is supported by our finding that HCV RNA contains m^6^A. While it is known that m^6^A functions on host mRNAs to regulate their stability, translation, localization, and interactions with RNA-binding proteins (Fu et al., 2014), we hypothesize that the function of m^6^A in HCV RNA is not due to regulation of HCV RNA stability or translation, because our studies of HCV RNA replication using a reporter virus found no change in reporter levels following depletion of the m^6^A machinery. Rather, our data suggest that m^6^A regulates infectious viral particle production through interactions of the viral RNA with host and viral proteins.

Because the writers (METTL3+14) and an eraser (FTO) of m^6^A regulated HCV particle production, it was reasonable to hypothesize that the m^6^A-binding YTHDF reader proteins would have a similar effect. All three YTHDF proteins bound to HCV RNA at similar sites and their depletion increased HCV particle production, suggesting that their effect on HCV particle production was due to binding HCV RNA (Figure 2; Table S2). Although YTHDF1 and YTHDF2 have been found to have divergent functions on host mRNAs, all three YTHDF proteins in our study acted similarly to suppress HCV (Wang et al., 2014, Wang et al., 2015). Likewise, during HIV-1 infection, all three YTHDF proteins function similarly to one another, although they have been described to have both pro- and anti-HIV function (Kennedy et al., 2016, Tirumuru et al., 2016). During HCV infection, YTHDF regulatory function is likely related to their relocalization to lipid droplets, the sites of viral assembly (Figure 3). Many RNA-binding proteins relocalize to lipid droplets in HCV-infected cells and regulate HCV particle production (Ariumi et al., 2011, Chatel-Chaix et al., 2013, Pager et al., 2013, Poenisch et al., 2015). Many of these proteins are known to interact with YTHDF proteins, suggesting that these interactions could regulate HCV particle production (Schwartz et al., 2014, Wang et al., 2015). Consequently, it will be important in the future to identify any YTHDF protein-protein interactions enriched during HCV infection, which may point to a regulatory network of RNA-binding proteins that affect infectious HCV particle production.

We found that about 50% of YTHDF protein-binding sites identified on HCV RNA using PAR-CLIP overlapped with MeRIP-seq m^6^A peaks (Figure 4). These results are similar to previous studies examining the overlap of YTHDF1 or YTHDF2 PAR-CLIP with MeRIP-seq data, which have found about a 60% overlap (Wang et al., 2014, Wang et al., 2015). We hypothesize that the non-overlapping YTHDF-binding sites in HCV RNA represent m^6^A sites not detected by MeRIP-seq due to biological variation, technical noise, or potentially sites that might be bound by YTHDF proteins in an m^6^A-independent fashion. A report found that YTHDF proteins bound to an in vitro transcribed, and hence non-methylated, HCV RNA genome (Ríos-Marco et al., 2016). Therefore, future studies could reveal functions of the YTHDF proteins that are independent of m^6^A during the HCV life cycle.

To discern the function of an m^6^A site on HCV RNA during infection, we abrogated m^6^A modification in the E1 region of HCV by mutation. This E1^mut^ virus produced higher viral titers than the WT virus (Figure 5), similar to what we found with METTL3+14 and YTHDF depletion and suggesting a conserved regulatory mechanism between both m^6^A and the YTHDF proteins at this site. The presence of these mutations in E1 increased HCV RNA binding to Core protein while reducing binding to YTHDF2. This suggests that interactions of the HCV RNA with Core are regulated by m^6^A such that viral genomes lacking m^6^A in the E1 region are preferentially segregated for packaging into nascent virions. Therefore, we hypothesize that the presence or absence of m^6^A in E1 facilitates competition between YTHDF protein and HCV Core binding to the viral genome, leading to the cellular retention or packaging of HCV RNA, respectively.

Because RNA viruses can rapidly evolve under selection pressure, the maintenance of m^6^A sites on the HCV genome suggests that m^6^A must confer an evolutionary advantage to the virus. In HCV, whose pathology is characterized by chronic progression during infection in the liver, a slower replication rate has been linked to persistent infection through an evasion of immune surveillance (Bocharov et al., 2004). Therefore, m^6^A may boost viral fitness by allowing HCV to establish slow, persistent infections. Pirakitikulr et al. (2016) identified a conserved stem loop in the E1 coding region, just downstream of our identified m^6^A sites, that suppresses viral particle production without affecting viral RNA replication. This raises the possibility that within the E1 region, multiple RNA elements, including m^6^A, play a role in segregating the RNA genome between stages of the HCV life cycle.

The function of the other m^6^A sites on the HCV RNA genome remains unknown. Because many of these sites do not overlap with YTHDF protein-binding sites, they may directly modify HCV RNA structure or recruit alternative m^6^A readers, such as HNRNPA1/B2, eIF3, or even METTL3 (Alarcón et al., 2015, Lin et al., 2016, Meyer et al., 2015). They may also contribute to antiviral innate immune evasion, because the presence of m^6^A on RNA has been shown to reduce its activation of toll-like receptor 3 signaling (Karikó et al., 2005). While we did not identify m^6^A in the known poly-U/UC pathogen-associated molecular patterns in the 3′ UTR of the HCV genome, we did find that YTHDF2 binds close to this region (Table S2), so future studies can begin to discern whether m^6^A plays a role in HCV innate immune evasion.

We found that four other *Flaviviridae* (DENV, YFV, ZIKV, and WNV) also contained m^6^A within their viral genomes. Because these viruses replicate in the cytoplasm, our data reveal that m^6^A methyltransferases are functional in the cytoplasm. Similar to the results of others, we detected the m^6^A machinery in cytoplasmic fractions (Figure S1C) (Chen et al., 2015, Harper et al., 1990, Lin et al., 2016). Therefore, cellular mRNAs could also be dynamically regulated by m^6^A modification following export into the cytoplasm. These viruses had prominent m^6^A peaks in NS5, which encodes their viral RNA-dependent RNA polymerase, strongly suggesting the presence of a conserved RNA regulatory element here. Both DENV and ZIKV (PR2015) contained m^6^A peaks within their envelope genes, similar to HCV, and future studies to determine whether these m^6^A sites also affect production of infectious flaviviral particles will be of interest. While the genomic RNA structures for DENV, YFV, ZIKV, and WNV have not yet been determined, these viral genomes contain specific RNA regulatory structures, especially within their UTRs. We found that two of the mosquito-transmitted viruses, DENV and YFV, have m^6^A within their 3′ UTRs (Figure 6F). In DENV, the 3′ UTR has two stem loops that regulate mosquito to human transmission (Villordo et al., 2015). Therefore, it is possible that m^6^A patterns and functionality in the mosquito-transmitted flaviviral genomes could contribute to vector-borne transmission. Finally, we observed clear differences in m^6^A patterns between the Dakar and the Puerto Rican isolates of ZIKV, which represent the African and the Asian lineages, respectively (Haddow et al., 2012). Because these lineages have differences in human disease, with increased pathogenicity ascribed to the Asian lineage of ZIKV (Weaver et al., 2016), the differences in regulation of these viruses by m^6^A could contribute to these varied infection outcomes.

In summary, we present global m^6^A profiling of RNA viruses within the *Flaviviridae* family. In addition, we provide evidence that an exclusively cytoplasmic RNA is modified by m^6^A. Furthermore, we present a role of this modification in regulating HCV RNA function at the level of infectious viral particle production. This work sets the stage to broadly study the role of m^6^A in *Flaviviridae* infection, transmission, and pathogenesis. This work also has the potential to uncover regulatory strategies to inhibit replication by these established and emerging viral pathogens.

## Experimental Procedures

### Cell Lines

Human hepatoma Huh7, Huh7.5, and Huh7.5 CD81 KO cells were grown in DMEM (Mediatech) supplemented with 10% fetal bovine serum (HyClone), 2 5 mM HEPES, and 1× non-essential amino acids (complete [c]DMEM; Thermo Fisher Scientific). HCV-K2040 (1B) replicon cells (Wang et al., 2003) were cultured in cDMEM containing 0.2 mg/mL geneticin (Thermo Fisher Scientific). The identity of the Huh7 and Huh7.5 cell lines was verified using the Promega GenePrint STR kit (DNA Analysis Facility, Duke University), and cells were verified as mycoplasma free by the LookOut Mycoplasma PCR detection kit (Sigma). Huh7.5 CD81 KO cells were generated by CRISPR, as described before, with details given in the Supplemental Experimental Procedures (Hopcraft et al., 2016, Hopcraft and Evans, 2015).

### Viral Infections and Generation of Viral Stocks

#### HCV

Infectious stocks of a cell culture-adapted strain of genotype 2A JFH1 HCV were generated and titrated by focus-forming assay (FFA), as described (Aligeti et al., 2015). HCV infections were performed at an MOI of 0.3 for 72 hr unless noted.

#### WNV

Working stocks of WNV isolate TX 2002-HC (WNV-TX) were generated in BHK-21 cells and titered as described (Suthar et al., 2010). WNV infections (MOI 5) were performed in Huh7 cells for 48 hr.

#### DENV and YFV

Preparation and titering of DENV2-NGC and YFV-17D stocks has been described (Le Sommer et al., 2012, Sessions et al., 2009). DENV and YFV infections (MOI 2) were performed for 24 hr in Huh7 cells.

#### ZIKV

ZIKV_PR2015 (PRVABC59) stocks were prepared and titered as described (Quicke et al., 2016). ZIKV_DAK (Zika virus/A.africanus-tc/SEN/1984/41525-DAK) stocks were generated and titered by FFA in Vero cells (Le Sommer et al., 2012). ZIKV infections (MOI 2) were performed in Huh7 cells for 24 hr.

### FFA for HCV Titer

Supernatants were collected and filtered through 0.45 μM syringe filters. Serial dilutions of supernatants were used to infect naive Huh7.5 cells in triplicate wells of a 48-well plate. At either 48 or 72 hpi, cells were fixed, permeabilized, and immunostained with HCV NS5A antibody (1:500; gift of Charles Rice, Rockefeller University). Following binding of horseradish peroxidase (HRP)-conjugated secondary antibody (1:500; Jackson ImmunoResearch), infected foci were visualized with the VIP Peroxidase Substrate Kit (Vector Laboratories) and counted at 40× magnification. Titer (in focus-forming units per milliliter) was calculated as described (Gastaminza et al., 2006). To measure intracellular HCV titer, cells pellets were washed in PBS, resuspended in serum-free media, and subjected to five rounds of freezing and thawing in a dry ice and ethanol bath. Lysate was cleared by centrifugation, and FFA was performed as described earlier.

### MeRIP-Seq

Poly(A)+ RNA purified from at least 75 μg total RNA (Poly(A) Purist Mag kit; Thermo Fisher Scientific) extracted from HCV-, DENV-, YFV-, WNV-, ZIKV (DAK)-, and ZIKV (PR2015)-infected samples was fragmented using the Ambion RNA fragmentation reagent and purified by ethanol precipitation. Fragmented RNA was heated to 75°C for 5 min, placed on ice for 3 min, and then incubated with anti-m^6^A antibody (5 μg; Synaptic Systems, #202111) conjugated to Protein G Dynabeads (50 μL; Thermo Fisher Scientific) in MeRIP buffer (50 mM Tris-HCl [pH 7.4], 150 mM NaCl, 1 mM EDTA, and 0.1% NP-40) overnight at 4°C. Beads were then washed 5× with MeRIP buffer, and bound RNA was eluted in MeRIP buffer containing 6.7 mM m^6^A sodium salt (Sigma). Eluted RNA was purified with the Quick-RNA miniprep kit (Zymo Research) and concentrated by ethanol precipitation. Sequencing libraries were prepared from this RNA, as well as input RNA, using the TruSeq RNA sequencing (RNA-seq) kit (Illumina). Libraries were sequenced to 1 × 50 base-pair reads on the Illumina HiSeq2500 at the Weill Cornell Medicine Epigenomics Core Facility. Reads were aligned to combined human (hg19) and viral genomes using Spliced Transcripts Alignment to a Reference (STAR), with a mapping quality threshold of 20. Despite the poly(A) enrichment, a significant number of reads mapped to the viral genomes. We identified peaks using MeRIPPeR (https://sourceforge.net/projects/meripper/), which defines peaks in m^6^A immunoprecipitation (IP) over input control read counts using Fisher’s exact test, with a minimum peak size of 100 bases. The false discovery rate (FDR) was set to <0.05 using a Benjamini-Hochberg correction. Intersections between the peaks called by two replicates provided the final set of peak calls. MeRIP-qRT-PCR followed this protocol, except that total RNA was not fragmented. Eluted RNA was reverse transcribed into cDNA and subjected to qRT-PCR.

### Statistical Analysis

Student’s unpaired t test and two-way ANOVA (with Bonferroni correction) were used for statistical analysis of the data using GraphPad Prism software. Graphed values are presented as mean ± SD (n = 3 or as indicated); ^∗^p ≤ 0.05, ^∗∗^p ≤ 0.01, and ^∗∗∗^p ≤ 0.001.

Additional experimental procedures can be found in the Supplemental Experimental Procedures.

## Author Contributions

N.S.G., A.B.R.M., M.J.M., A.E.R., E.M.K., C.E.M., and S.M.H. designed experiments and analyzed the data. N.S.G., A.B.R.M., M.J.M., E.M.K., A.E.R., C.V., J.W., J.A.G., S.E.H., K.M.Q., B.A.L., O.R.I., S.B.B., and S.M.H. performed the experiments. C.L.H., M.J.E., M.S.S., and M.A.G.-B. provided reagents. N.S.G., A.B.R.M., C.E.M., and S.M.H. wrote the manuscript. All authors contributed to editing.

## Accession Numbers

The accession number for the raw sequencing data obtained from the MeRIP-seq and PAR-CLIP and reported in this paper is GEO: GSE83438.

## Supplemental Information

Document S1. Supplemental Experimental Procedures, Figures S1–S6, and Tables S1–S3

Document S2. Article plus Supplemental Information
