# Supplementary material for: N6-Methyladenosine in Flaviviridae Viral RNA Genomes Regulates Infection
Source: Cell Host Microbe. 2016 Nov 9;20(5):654–65. doi: 10.1016/j.chom.2016.09.015 (PMC5123813; doi:10.1016/j.chom.2016.09.015)
Supplement: Document S1. Supplemental Experimental Procedures, Figures S1–S6, and Tables S1–S3 [file mmc1.pdf]

Cell Host & Microbe, Volume *20*

## Supplemental Information

# *N6*-Methyladenosine in *Flaviviridae* Viral RNA Genomes Regulates Infection

**Nandan S. Gokhale, Alexa B.R. McIntyre, Michael J. McFadden, Allison E. Roder, Edward M. Kennedy, Jorge A. Gandara, Sharon E. Hopcraft, Kendra M. Quicke, Christine Vazquez, Jason Willer, Olga R. Ilkayeva, Brittany A. Law, Christopher L. Holley, Mariano A. Garcia-Blanco, Matthew J. Evans, Mehul S. Suthar, Shelton S. Bradrick, Christopher E. Mason, and Stacy M. Horner**

**SUPPLEMENTAL INFORMATION**

Table of contents:

Figure S1. Related to Figures 1 and 4.

Figure S2. Related to Figure 2.

Figure S3. Related to Figure 3

Figure S4. Related to Table S2

Figure S5. Related to Figure 5.

Figure S6. Related to Figure 4 and Figure 6.

Table S1. Related to Figure 4

Table S2. Related to Figure 4.

Table S3. Related to Figure 6.

Supplemental experimental procedures

Supplemental references

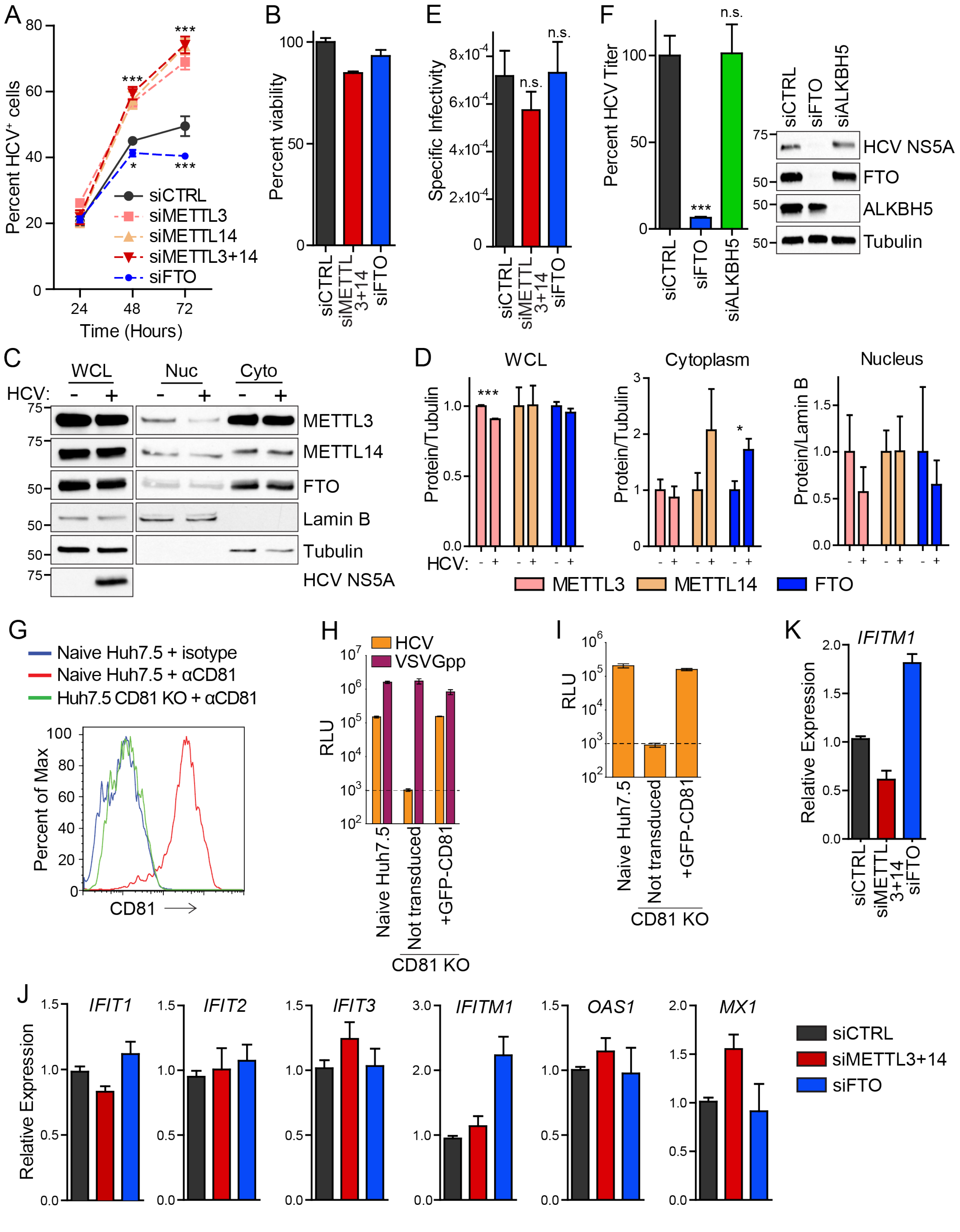


**Figure S1. Related to Figures 1 and 4.** (A) Quantification of the percent of HCV-infected Huh7 cells at the indicated time points as determined by immunofluorescence staining of HCV NS5A protein of Huh7 cells following

treatment with the indicated siRNAs. Values are presented as mean ± SD (n=3), with ≥5000 cells counted per condition. **$p \leq 0.05$*, ****$p \leq 0.001$* by two-way ANOVA with Bonferroni correction. (B) Viability of HCV-infected Huh7 cells treated with indicated siRNAs as measured at 72 hpi by Cell-Titer GLO assay. Data are presented as mean ± SD (n=3). (C) Immunoblot analysis of isolated nuclear (Nuc) and cytoplasmic (Cyto) biochemical fractions, as well as whole cell lysate (WCL), from extracts of mock- (-) or HCV- infected (+) Huh7 cells. (Lamin B – nuclear marker; Tubulin – cytoplasmic marker). (D) Quantification of protein levels in WCL (relative to Tubulin), or isolated nuclear (relative to Lamin B) and cytoplasmic (relative to Tubulin) fractions from extracts of mock- (-) or HCV-infected (+) Huh7 cells. Data are presented as the mean ± SEM of three independent experiments. (E) Specific infectivity of HCV particles produced by Huh7 cells treated with the indicated siRNAs. Specific infectivity was calculated as a ratio of HCV titer over HCV RNA isolated from the supernatant, measured at 72 hpi. Data are presented as the mean ± SEM of three independent experiments. (F) Focus forming assay of supernatants harvested from Huh7 cells at 72 hpi after treatment with the indicated siRNAs. Data are presented as the mean ± SEM of three independent experiments. (Right panel) Representative immunoblot analysis of extracts of HCV-infected Huh7 cells treated with the indicated siRNAs. (G) Parental Huh7.5 cells or Huh7.5 CD81 KO cells were stained with negative isotype control or CD81-specific antibodies and analyzed by fluorescence associated cell sorting. (H) Cells in (G) were infected with *Gaussia* luciferase expressing HCV Jc1 virus (orange bars) or vesicular stomatitis virus glycoprotein bearing lentiviral particles (VSVGpp) (purple bars) at an MOI of 0.05. Luciferase values are expressed as relative luciferase units (RLU). Data are presented as mean ± SEM of two independent experiments, each performed in triplicate (I) Naïve Huh7.5 cells were infected with supernatants from the HCV infections of the infections shown in panel (H). (J) RT-qPCR analysis of the indicated interferon stimulated genes relative to the housekeeping gene *RPL30* from RNA extracted from Huh7 cells treated with the indicated siRNAs at 48 hours post HCV-infection. (K) RT-qPCR analysis of *IFITM1* expression relative to *RPL30* from RNA extracted from Huh7 cells treated with the indicated siRNAs at 24 hpi. Data in (J) and (K) are presented as mean ± SEM of two independent experiments performed in triplicate. **$p \leq 0.05$*, ****$p \leq 0.001$* by unpaired Student's *t*-test.

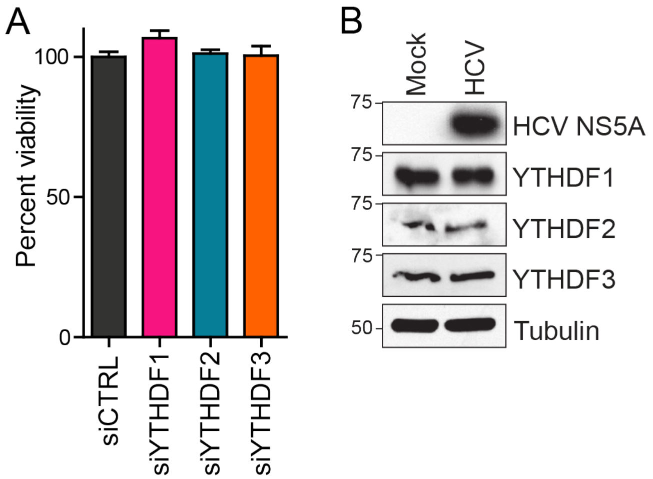


**Figure S2. Related to Figure 2.** (A) Viability of HCV-infected Huh7 cells treated with indicated siRNAs as measured at 72 hours post HCV-infection by Cell-Titer GLO assay. Data are presented as mean ± SD (n=3). (B) Immunoblot analysis of YTHDF protein expression in mock- and HCV infected Huh7 cells.

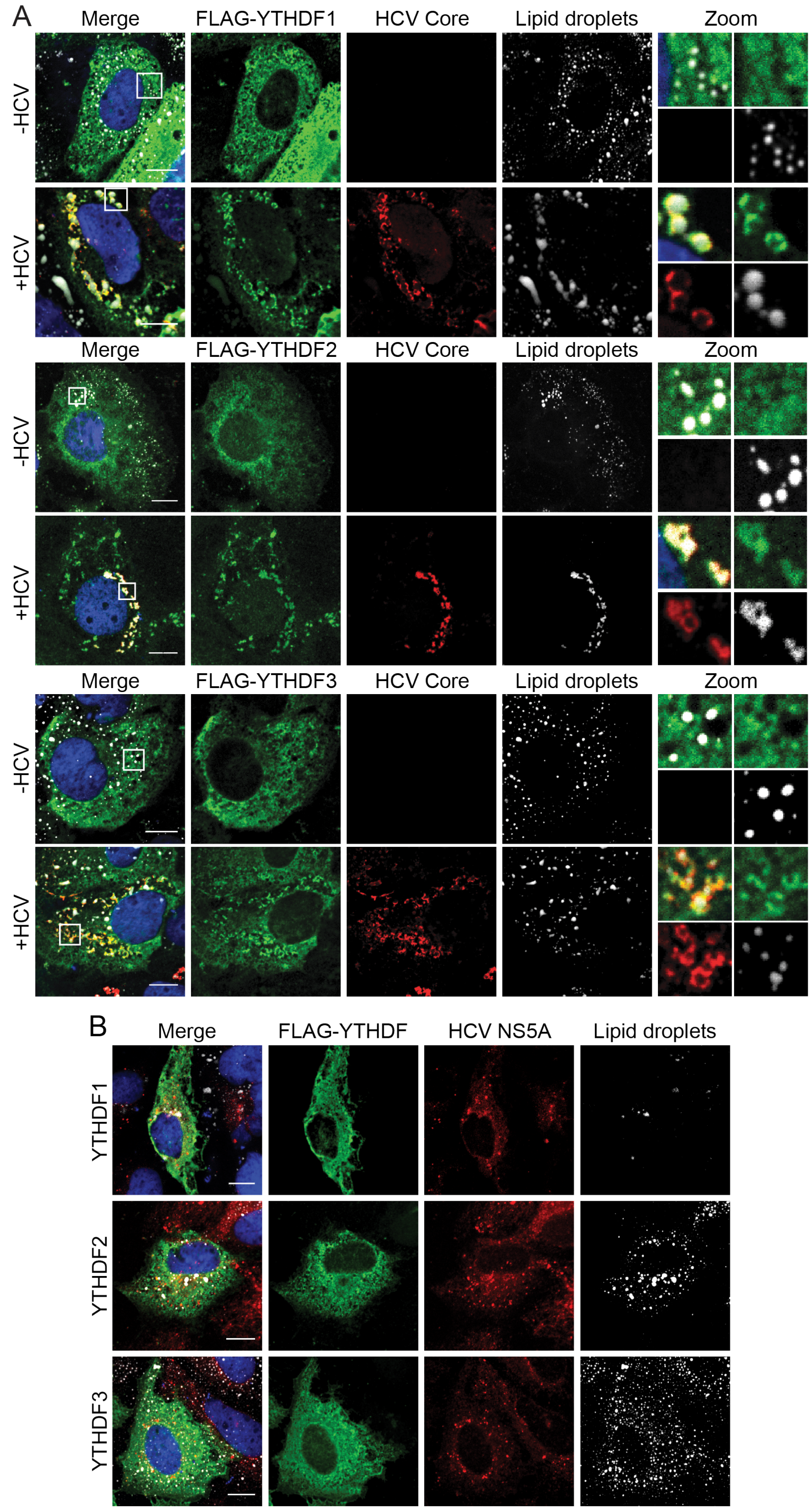
A
Merge
FLAG-YTHDF1
HCV Core
Lipid droplets
Zoom
-HCV
+HCV
Merge
FLAG-YTHDF2
HCV Core
Lipid droplets
Zoom
-HCV
+HCV
Merge
FLAG-YTHDF3
HCV Core
Lipid droplets
Zoom
-HCV
+HCV
B
Merge
FLAG-YTHDF
HCV NS5A
Lipid droplets
YTHDF1
YTHDF2
YTHDF3

**Figure S3. Related to Figure 3.** (A) Confocal micrographs of mock- or HCV-infected Huh7 cells expressing the indicated FLAG-tagged YTHDF proteins, immunostained with anti-FLAG (green) and anti-HCV core (red). Lipid droplets (grey) and nuclei (blue) were labeled with BODIPY and DAPI, respectively. (B) Huh7 cells stably replicating an HCV subgenomic replicon (Genotype 1B, K2040), and expressing the indicated FLAG-tagged YTHDF proteins and stained as in (A). Single cell images are representative of >30 cells analyzed. Scale bar, 10 μm.

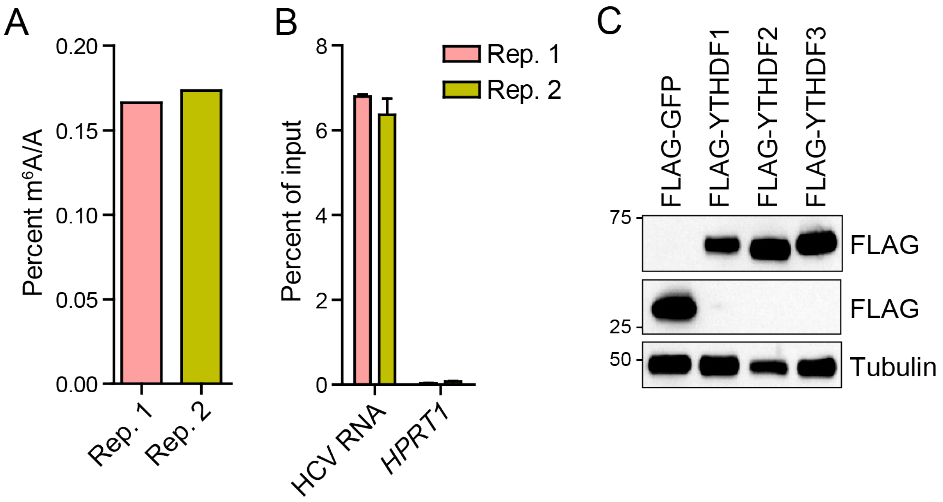


**Figure S4. Related to Figure 4 and Table S2.** (A) The ratio of $m^6A/A$ by UPLC-MS/MS in HCV RNA purified from HCV-infected Huh7 cells by using biotinylated antisense oligonucleotides. Rep. 1 and Rep. 2 indicate two independent experiments. (B) RT-qPCR analysis of purified HCV RNA used in (A) demonstrating the specificity of the antisense oligonucleotide pulldown to HCV RNA but not the control RNA *HPRT1*. (C) Immunoblot of Huh7 cell lines stably expressing FLAG-tagged YTHDF proteins or FLAG-GFP used for PAR-CLIP analysis.

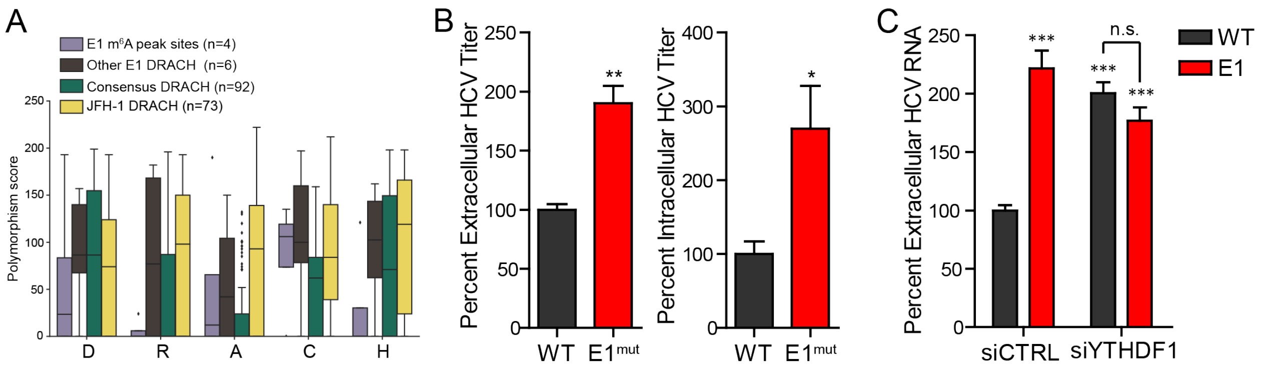


**Figure S5. Related to Figure 5.** (A) An analysis of single nucleotide polymorphism (SNP) rates per base showed DRACH sequences found in the JFH1 genome and in the consensus between 26 genomes (obtained: https://euhcvdb.ibcp.fr/euHCVdb/jsp/nomen_tab1.jsp) representing all HCV genotypes ("Consensus DRACH") had lower polymorphism scores than DRACH sites found in the JFH1 reference strain but not the consensus ("JHF1 DRACH"). The A residue was particularly low scoring ($p=3.00x10^{-11}$) in a *t*-test between Consensus DRACH and JHF1 DRACH, suggestive of conserved methylation. Scores for the R, C, and H residues were also significantly lower ($p=3.95x10^{-5}$*, 2.84x*$10^{-5}$*, and 0.02,* respectively). The set of four DRACH motif sites proximal to the E1 $m^6A$ peak we identified from MeRIP-seq data ("E1 $m^6A$ peak sites") showed generally lower scores than the JFH1 DRACH, although only the R and H positions were statistically significant (*p=0.02* and *p=0.04*). A control set of all other DRACH motifs found in the E1 region of the reference strain ("Other E1 DRACH") showed no significant differences in SNP rates with the JFH1 DRACH. We calculated polymorphism scores after a multiple sequence alignment using MUSCLE, where the score per position = -100*sum(p*log(p)), and p = frequency of each A, C, G, or T as described by ViPR (http://www.viprbrc.org). "Consensus" indicates that each position in the alignment contains a base consistent with the motif in > 50% of sequences. Statistical significance was determined by Student's *t*-test. (B) Focus-forming assay of extracellular and intracellular virus harvested from Huh7 cells after electroporation of WT or $E1^{mut}$ HCV RNA (48h). Data are analyzed as the percent of viral titer relative to WT, and are presented as the mean ± SEM from at least four independent experiments. (C) Viral RNA in the supernatant harvested from Huh7 cells after treatment with the indicated siRNAs and infection with WT or $E1^{mut}$ HCV, as measured by RT-qPCR. Data are presented as the mean ± SD, and are representative of two experiments done in triplicate. *$p \leq 0.05$, *$*\leq 0.01$, p***$p \leq 0.001$* by unpaired Student's *t*-test relative to siCTRL-WT sample.

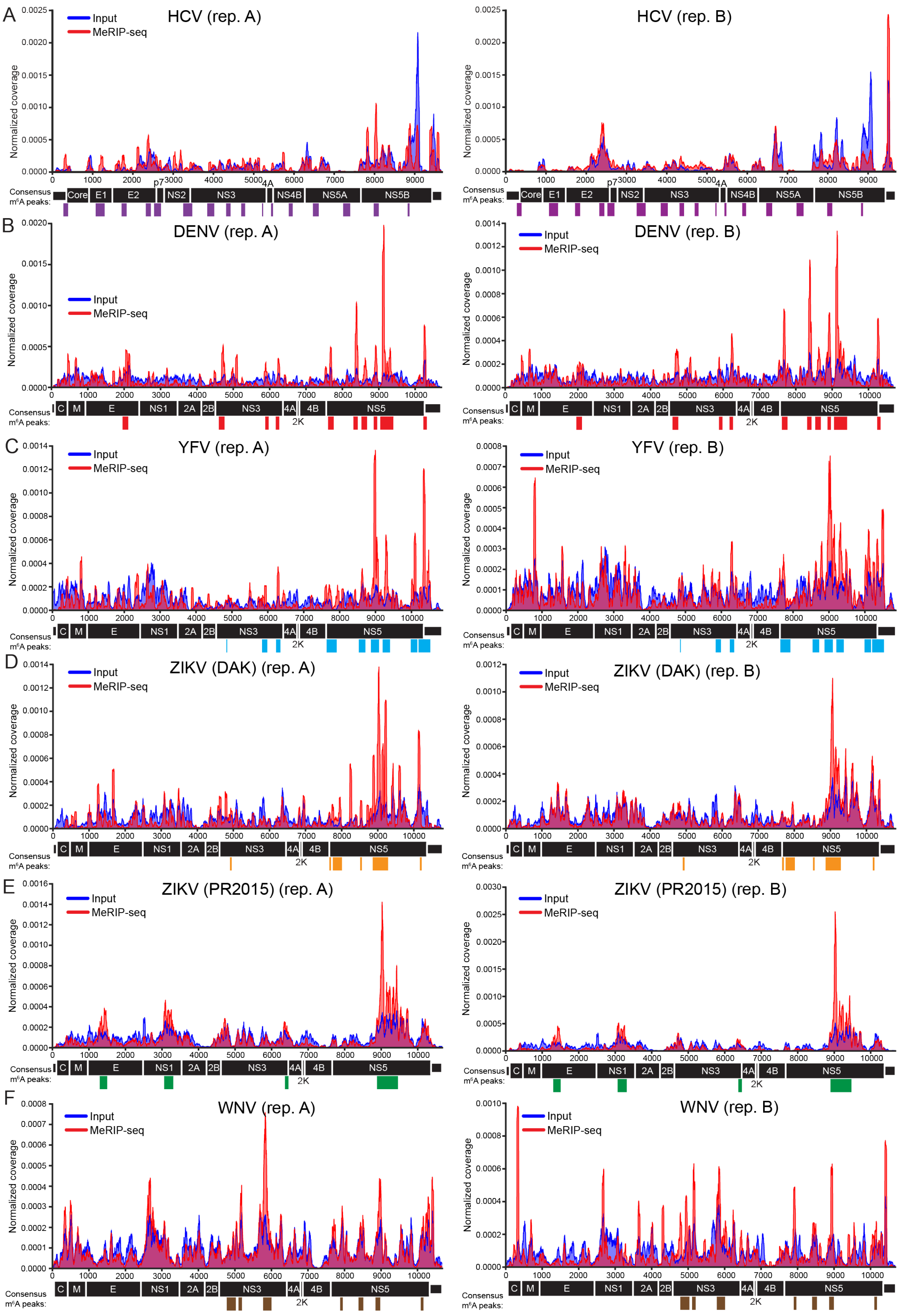
A
HCV (rep. A)
HCV (rep. B)
Input
MeRIP-seq
Normalized coverage
Consensus m6A peaks:
Core
E1
E2
p7
NS2
NS3
4A
NS4B
NS5A
NS5B
B
DENV (rep. A)
DENV (rep. B)
C
M
E
NS1
2A
2B
NS3
4A
4B
2K
NS5
C
YFV (rep. A)
YFV (rep. B)
D
ZIKV (DAK) (rep. A)
ZIKV (DAK) (rep. B)
E
ZIKV (PR2015) (rep. A)
ZIKV (PR2015) (rep. B)
F
WNV (rep. A)
WNV (rep. B)

**Figure S6. Related to Figure 4 and Figure 6.** Maps from duplicate MeRIP-seq experiments used to identify $m^6A$ sites on the genomes of the Flaviviridae members (A) HCV (B) DENV, (C) YFV, (D) ZIKV (DAK), (E) ZIKV (PR2015), and (F) WNV. MeRIP-seq coverage is plotted in red, and input RNA-seq coverage in blue. Colored bars indicate $m^6A$ peaks identified in two independent experiments by MeRIPPeR analysis. (FDR-corrected q-value <0.05). "Rep. A" maps are identical to those displayed in Figures 4 and 6, and are shown here for comparison between replicates.

| Feature | $m^6A$ Peak (nt) |
|---|---|
| 5'UTR | 275-340 |
| Core | 341-400 |
| E1 | 1050-1275 |
| E2 | 1700-1825 |
| | 2300-2425 |
| | 2550-2587 |
| p7 | 2588-2725 |
| NS2 | 3250-3430 |
| NS3 | 3431-3475 |
| | 3850-4025 |
| | 4325-4425 |
| | 4675-4800 |
| | 5200-5225 |
| NS4A | 5425-5475 |
| NS4B | 5825-5925 |
| NS5A | 6450-6600 |
| NS5B | 7200-7375 |
| | 7975-8100 |
| | 8750-8800 |

**Table S1**. **Related to Figure 4. $m^6A$ sites on the HCV RNA genome as determined by MeRIP-seq.** Peaks called by intersecting MeRIPPeR analyses of two independent experiments. Numbering corresponds to the JFH-1 strain of HCV (GenBank accession: AB047639). Nineteen peaks were identified.

| Feature | YTHDF1 | YTHDF2 | YTHDF3 | DRA$^{m}$CH motif? |
|---|---|---|---|---|
| 5'UTR | 171-199 | 163-199 | | Yes |
| Core | 590-627 | 590-627 | 602-627 | Yes |
| | | 746-769 | | |
| E1 | 1262-1283 | 1267-1290 | | Yes |
| E2 | | 1821-1838 | | |
| | 1994-2020 | 1998-2014 | | Yes |
| | | | 2045-2089 | Yes |
| | 2181-2211 | 2181-2211 | 2181-2211 | Yes |
| | | 2305-2322 | | Yes |
| | | 2336-2356 | | |
| | | | 2454-2473 | |
| p7 | | 2600-2618 | | |
| | 2728-2761 | | 2719-2742 | |
| NS2 | 3349-3366 | | | Yes |
| NS3 | | | 3640-3646 | |
| | | | 3745-3762 | |
| | 3922-3937 | | | |
| | | 4675-4712 | | Yes |
| | | 4741-4765 | | Yes |
| | | 4818-4851 | | Yes |
| NS4A | 5391-5410 | | | |
| | | | 5433-5476 | |
| NS4B | | 5753-5792 | | |
| NS5A | | 6321-6349 | | Yes |
| | 6362-6384 | 6362-6384 | | |
| | | 6450-6467 | | |
| | 6791-6815 | 6774-6815 | 6803-6827 | |
| | | 6842-6865 | | Yes |
| | | 6909-6944 | | |
| | | 6968-7008 | | |
| | 7542-7573 | 7540-7573 | | Yes |
| NS5B | | | 7764-7807 | Yes |
| | | 7870-7897 | | Yes |
| | | 8157-8183 | | Yes |
| | 8429-8464 | 8443-8464 | | Yes |
| | | 8688-8717 | 8696-8716 | Yes |
| | 8826-8852 | | 8828-8852 | Yes |
| | | | 8866-8908 | Yes |
| | 8913-8944 | | | Yes |
| | 9266-9289 | | 9266-9304 | Yes |
| | | | 9317-9334 | |
| | | 9376-9407 | | |

**Table S2. Related to Figure 4. YTHDF protein binding sites as determined by PAR-CLIP.** Peaks called by PARalyzer that map to the same region are displayed in the same row. Numbering corresponds to JFH1 strain of HCV (GenBank accession: AB047639). The presence of DRA$^{m}$CH motifs within YTHDF protein binding sites is indicated.

| Feature | $m^6A$-peaks (nt) | | | | |
|---|---|---|---|---|---|
| | DENV | YFV | ZIKV (DAK) | ZIKV (PR2015) | WNV |
| E | 1975-2125 | | | 1300-1500 | |
| NS1 | | | | 3050-3300 | |
| NS3 | 4650-4800 | 4850-4875 | 4850-4900 | 6350-6450 | 4775-5025 |
| | 5825-5929 | 5825-5975 | | | 5100-5200 |
| | 6200-6300 | 6225-6350 | | | 5775-6000 |
| NS4B | | | 7625-7666 | | |
| NS5 | 7650-7800 | 7675-7950 | 7667-7675 | 8900-9475 | 7875-7950 |
| | 8325-8450 | 8575-8750 | 7750-8000 | | 8400-8525 |
| | 8550-8700 | 8875-9100 | 8675-8725 | | 8850-8975 |
| | 8875-8975 | 9200-9400 | 8850-9275 | | 10100-10175 |
| | 9075-9375 | 9975-10150 | 10125-10175 | | |
| | 10200-10272 | 10225-10354 | | | |
| 3'UTR | 10273-10300 | 10355-10550 | | | |

**Table S3. Related to Figure 6. $m^6A$ sites on the RNA genomes of the indicated virus as determined by MeRIP-seq.** Peaks were called by intersecting MeRIPPeR analyses of two independent experiments. Numbering corresponds to the DENV2-NGC (Genbank: KM204118.1), YFV-17D (Genbank: NC_002031.1), ZIKV (DAK) (Genbank: KU955591.1), ZIKV (PR2015) (Genbank: KU501215.1), and WNV-TX (Genbank: DQ176637.1) genomes.

## SUPPLEMENTAL EXPERIMENTAL PROCEDURES

**Plasmids.** These plasmids have been described previously: pJFH1-QL/GLuc2A (Yamane et al., 2014), psJFH1-p7+NS5B, which contains cell culture adaptive mutations in p7 (C766Y) and NS5B (R2676K) (Aligeti et al., 2015), and pLEX-FLAG-YTHDF1, 2, 3 (Kennedy et al., 2016). The following plasmids were constructed in this study: pEF-Tak-Flag-YTHDF1, 2, 3; psJFH1-p7+NS5B-E1$^{mut}$, pJFH1-QL/GLuc2A-E1$^{mut}$, psJFH1-p7+NS5B-GNN; psiCHECK2-HCV WT or HCV E1$^{mut}$; and pSgCD81_1 and pSgCD81_2. All DNA sequences were verified by sequencing. FLAG-tagged YTHDF constructs were generated by cloning PCR amplified products (YTHDF1, MGC cloneID-BC050284; YTHDF2, MGC cloneID-BC002559; YTHDF3, MGC cloneID-BC052970) into the pEF-Tak expression vector using *Not*I and *Pme*I (Saito et al., 2007). psJFH1-p7+NS5B-E1$^{mut}$ (with nucleotide mutations A1285T, C1303T, C1315T, and C1378T) was generated by inserting a synthesized gBlock with the desired mutations (nt972-1749, Integrated DNA Technologies) into a pCR-TOPO holding vector containing the JFH1 sequence (nt97-4333), and mutant *AgeI-KpnI* digested fragment was then subcloned into psJFH1-p7+NS5B. A similar strategy using *AgeI* and *KpnI* was used to clone the pJFH1-QL/GLuc2A-E1$^{mut}$ reporter plasmid. psJFH1-p7+NS5B-GNN was generated by site-directed mutagenesis to insert the lethal NS5B D318N and D319N mutations in a pCR-TOPO holding vector containing the psJFH1-p7+NS5B region between nt8155 and the spectinomycin resistance gene, and the *HindIII-XbaI* fragment subcloned into psJFH1-p7+NS5B. psiCHECK2-HCV WT or HCV E1$^{mut}$ was generated by amplifying nt1233-1439 of JFH1 by PCR then subcloning into the *XhoI-NotI* sites of the psiCHECK22 vector (Promega). All nucleotide and amino acid positions refer to the JFH1 genome (GenBank accession number: AB047639).

For CRISPR-mediated gene knockout, we generated two expression plasmids encoding U6 promoter driven human CD81-specific guide RNAs (gRNAs) (Mali et al., 2013). Two rounds of overlapping PCR were performed by amplifying a gRNA encoding plasmid (provided by George Church, Harvard University; Addgene plasmid # 41819): in the first round, PCR products were generated encompassing the U6 promoter through the 5′ end of the gRNA (consisting of the specific target sequence) with the ME-O-1122 oligonucleotide (5′-CGGGCCCCCCCTCGAGTGTACAAAAAGCAGGCT) and a CD81 target sequence specific reverse oligonucleotide (see list below). A second PCR product was generated encompassing a region from the CD81 target sequence through the end of the gRNA coding sequence with a forward direction CLDN1 target sequence specific oligonucleotide (ME-O-1138; 5'-GCTTCATTCTCGCCTTCCGTTTTAGAGCTAGAAATA). These products were then reamplified with only the outer oligonucleotides, ME-O-1122 and -1123, to produce single PCR products flanked by *XhoI* and *EcoRI* sites at the 5′ and 3′ ends, respectively, and cloned into pBlueScript. Two separate CD81-specific gRNA plasmids were created with the following forward and reverse oligonucleotide combination: ME-O-1252/1251; 5'-GGGCTGCTACGGGGCCATC CGGTGTTTCGTCCTTTCC / 5'-GATGGCCCCGTAGCAGCCC GTTTTAGAGCTAGAAATA, and ME-O-1254/1253; 5'-TCTCGTGGAAGGTCTTCAC CGGTGTTTCGTCCTTTCC / 5'-CACCTGTACACGTAGGGCC GTGAAGACCTTCCACGAGA GTTTTAGAGCTAGAAATA, which target nucleotides 467-485 and 672-690 of the CD81 human cDNA (Genbank accession number: NM_004356), respectively. The sequences of oligonucleotide primers and plasmids used are available upon request.

***In vitro* transcription of HCV RNA and electroporation.** Plasmid DNA encoding the described HCV constructs was linearized using *XbaI*. Linearized DNA treated with proteinase K (Thermo-Fisher) and Mung bean nuclease (NEB) was purified by phenol-chloroform extraction and was used as a template for *in vitro* transcription with a MEGAscript T7 Transcription Kit (Thermo-Fisher). Following DNase treatment, RNA was purified by phenol-chloroform extraction and run on a denaturing gel to ensure integrity. For electroporation, 5µg of *in vitro* transcribed viral RNA was electroporated into 4x10$^6$ Huh7.5 cells at 250V and 950µF with a Gene Pulser Xcell system (Bio-Rad).

**Luciferase assays for HCV Replication.** Huh7.5 CD81 KO cells cultured in 12-well plates were transfected with specific siRNAs. At 24 hours after siRNA treatment, 1µg of *in vitro* transcribed JFH1-QL/GLuc2A reporter HCV RNA was transfected into cells using the *Trans*IT-mRNA transfection kit (Mirus Bio), according to the manufacturer's instructions. *Gaussia* luciferase activity in supernatant harvested at 4, 24, 48 and 72 hours post-transfection was measured using the BioLux *Gaussia* Luciferase assay kit (NEB).

**Quantification of RNA by RT-qPCR.** RNA was reversed transcribed using the iScript cDNA synthesis kit (Bio-Rad) as per manufacturer's instructions, and synthesized cDNA was diluted 1:5 in water. RT-PCR was performed in triplicate with the Power SYBR Green PCR master mix (Thermo-Fisher) using the Applied Biosystems Step One Plus RT-PCR system. The sequences of primers used for RT-qPCR are:

| Primer Name | Primer Sequence |
|---|---|
| HCV 5'UTR | S-AGAGCCATAGTGGTCTGCGG |
| | AS-CTTTCGCAACCCAACGCTAC |
| HCV E1 | S-ATCTACCCTGGCACCATCAC |
| | AS-AGTAGGCCAAGCCGAACAT |
| *SON* | S-TGACAGATTTGGATAAGGCTCA |
| | AS-GCTCCTCCTGACTTTTTAGCAA |
| *HPRT1* | S-TGACACTGGCAAAACAATGCA |
| | AS-GGTCCTTTTCACCAGCAAGCT |

To measure extracellular HCV RNA, viral RNA from filtered cell supernatants was extracted using a QIAamp viral RNA kit (Qiagen), as recommended by the manufacturer. HCV RNA copy number was measured in triplicate by RT-qPCR using the TaqMan Fast Virus 1-Step Mix (Qiagen) with an HCV-specific probe targeting the 5' untranslated region of HCV (Assay ID: Pa03453408_s1). The copy number of HCV was calculated by comparison to a standard curve of a full-length *in vitro* transcribed HCV RNA, as described (Aligeti et al., 2015).

**Immunofluorescence analysis and confocal microscopy.** Cells were fixed in 4% paraformaldehyde in PBS, permeabilized with 0.2% Triton X-100 in PBS, and blocked with 3% BSA in PBS. Slides were stained with antibodies against YTHDF1 (Proteintech, 1:100), YTHDF2 (Proteintech, 1:100), YTHDF3 (Sigma, 1:100), or FLAG (Sigma; 1:1000), and HCV core protein (Thermo-Fisher; 1:500), washed 3x with PBS, and stained with conjugated AlexaFluor secondary antibodies (Life Technologies), BODIPY 493/503 (Invitrogen; 1:150) along with DAPI (1:200, Life Technologies), and mounted with ProLong Gold (Invitrogen). Imaging was performed on a Zeiss 710 laser scanning confocal microscope, or a Leica SP5 inverted confocal microscope using a 63x/1.25 oil objective using 405, 488, 561 and 633 laser lines at a 4x optical zoom with pinholes set to 1 AU for each channel (Light Microscopy Core Facility, Duke University). Gain and offset settings were optimized and final images were taken with line averaging of 4. All images were processed with NIH Fiji/ImageJ (Schindelin et al., 2012).

The enrichment index of YTHDF proteins measures the enrichment of YTHDF staining in the area proximal to lipid droplets, relative to that in the area distal to lipid droplets. This was calculated by using NIH Fiji/ImageJ to measure the mean brightness of YTHDF proteins in an area of 0.4μM around lipid droplets, as identified by BODIPY staining. Then, the YTHDF mean brightness in an area around the lipid droplets expanded by a further 0.4μM (between 0.4μM and 0.8μM) was subtracted from the first value.

**Immunoblotting.** Cells were lysed in a modified RIPA buffer (10mM Tris [pH 7.5], 150mM NaCl, 0.5% sodium deoxycholate, and 1% Triton X-100) supplemented with protease inhibitor cocktail (Sigma) and phosphatase inhibitor cocktail II (Millipore), and post-nuclear lysates were harvested by centrifugation. Quantified protein (between 5-15μg) was resolved by SDS/PAGE, transferred to nitrocellulose membranes in a 25 mM Tris-192 mM glycine-0.01% SDS buffer and blocked in StartingBlock (Thermo-Fisher) buffer. After washing with PBS-T buffer, membranes were incubated with species-specific horseradish peroxidase-conjugated antibodies (Jackson ImmunoResearch, 1:5000) followed by treatment of the membrane with ECL+ (GE Healthcare) and imaging on X-ray film. The following antibodies were used for immunoblot: anti-METTL3 (Novus Biologicals, 1:5000), anti-METTL14 (Sigma, 1:5000), anti-FTO (Abcam, 1:1000), anti-YTHDF1 (Abcam, 1:1000), anti-YTHDF2 (Santa Cruz Biotechnology, 1:500), anti-YTHDF3 (Sigma, 1:1000), anti-ALKBH5 (Sigma, 1:1000), anti-FLAG M2 (Sigma, 1:5000), anti-tubulin (Sigma, 1:5000), anti-HCV NS5A (gift of Charles Rice, 1:1000), and anti-HCV Core (Thermo-Fisher, 1:500).

**RNA-immunoprecipitation.** Cell extracts were harvested in polysome lysis buffer (100mM KCl, 5mM $MgCl_2$, 10mM HEPES (pH 7.0), 0.5% Nonidet P-40 (NP-40)) supplemented with protease inhibitor cocktail (Sigma) and RNaseIN ribonuclease inhibitor (Promega), and lysates were cleared by centrifugation. RNP complexes were immunoprecipitated with anti-FLAG antibody conjugated to magnetic beads (Sigma) overnight at 4°C with head-over-tail rotation, and then washed five times in ice-cold NT2 buffer (50mM Tris-HCl (pH 7.4), 150mM NaCl, 1mM $MgCl_2$, 0.05% NP-40). Protein for immunoblotting was eluted from ten percent of beads by boiling in 2X Laemmli sample buffer (Bio-Rad). RNA was extracted from ninety percent of beads using TRIzol reagent (Thermo-

Fisher). Equal volumes of eluted RNA were used for cDNA synthesis, quantified by RT-qPCR and normalized to RNA levels in input samples. For HCV Core RIP, a similar procedure was employed except that RNP complexes were purified with Protein G Dynabeads conjugated to anti-Core.

For immunoprecipitation of reporter RNAs by FLAG-YTHDF2, Huh7 cells were co-transfected with WT or $E1^{mut}$ psiCHECK2 plasmids along with plasmid expressing FLAG-YTHDF2 or vector control for 48 hours and RNA-immunoprecipitation was performed on extracts as described above.

**siRNA and siRNA transfections.** siRNAs directed against METTL3 (SI04317096), METTL14 (SI00459942), FTO (SI04177530), ALKBH5 (SI04138869), YTHDF1 (SI00764715), YTHDF2 (SI04174534), YTHDF3 (SI04205761), or non-targeting AllStars negative control siRNA (1027280) were purchased from Qiagen. All siRNA transfections were performed using the Lipofectamine RNAiMAX transfection reagent (Thermo-Fisher) according to manufacturer's instructions.

**Generation of Huh7.5 CD81 KO cells by CRISPR.** Huh7.5 cells were transiently transfected with expression plasmids encoding a human codon optimized Cas9 protein from *Streptococcus pyogenes* (provided by G. Church, Harvard, Addgene plasmid # 41815) (Mali et al., 2013) and a pooled mixture of the CD81 gRNA expression plasmids (pSgCD81_1 and pSgCD81_2). Transfected cells were passaged for one to two weeks to allow the turnover of previously translated target protein. Cells were then FACS sorted for loss of CD81 following staining with the anti-CD81 antibody (mAb JS81, BD Pharmingen) and a goat anti-human Alexa-647 antibody (Thermo-Fisher). Knockout efficiency prior to sorting was between 7-8%. CD81 negative cells were then FACS sorted following staining with the same antibodies, and single cell clones were derived by dilution cloning in 96-well plates. Individual clones were expanded and assayed for CD81 expression by FACS staining, and their capacity to support HCV infection by limited dilution assay based on NS5A staining, as previously described (Meuleman et al., 2012). We chose Huh7.5 CD81 KO clone 5 as a representative single cell clone for further experiments. These cells, like all CD81 KO cells examined, lacked CD81 expression (Fig. S1G) and could neither be infected with (Fig. S1H) or subsequently produce infectious HCV (Fig. S1I).

**PAR-CLIP.** FLAG-YTHDF or FLAG-GFP Huh7 cells lines were generated using pLEX-based lentiviral vectors as described (Kennedy et al., 2016), followed by clonal isolation. $6x10^7$ cells from each cell line were seeded and the next day infected with HCV (MOI 0.2). 3 hpi, cells were pulsed with 100µM 4SU in fresh media. 48 hpi, cells were cross-linked (UV 365nm), harvested and PAR-CLIP was performed as described (Hafner et al., 2010; Kennedy et al., 2016). PAR-CLIP libraries were sequenced on a HiSeq 2000, base calling was performed with CASAVA, and data was then processed with the fastx toolkit (http://hannonlab.cshl.edu/fastx_toolkit/). Reads with a length greater than 15 bp were used for downstream bioinformatic analysis. All alignments were performed with Bowtie (Langmead et al., 2009). Reads were initially aligned to the human genome build hg19 allowing up to 1 mismatch, and unaligned reads were then aligned to the HCV genotype 2A JFH1 cell culture-adapted viral genome (Aligeti et al., 2015) allowing 2 mismatches. These reads were then analyzed with PARalyzer (Corcoran et al., 2011) with a minimum cluster read depth of 3; the set of FLAG-GFP clusters (background) were then subtracted from the FLAG-YTHDF clusters with in house Perl scripts to yield the final dataset.

**Viral RNA purification and LC-MS.** 100µg total RNA from HCV-infected Huh7 cells was mixed with 200pmol each of five 5'-biotinylated oligonucleotides which were antisense to HCV RNA (Integrated DNA Technologies) in a total volume of 120µl, heated to 90°C for 5 minutes, and allowed to cool to room temperature. Oligonucleotide sequences are : 5' – GGTGCACGGTCTACGAGACCTCCC, 5' – CACGGACCTTTCACAGCTAGCCGT, 5' – GGGATCTCACCCTCCCGCCCGAGG, 5' – TAACGATGTCTATGATGACCTC, 5' – CAGAAGGATGACAATGACCTTC). Streptavidin MyOne C1 beads (100µl; Thermo-Fisher) were washed as recommended by the manufacturer, resuspended in 120µl 2X B&W buffer (10mM Tris-HCV, 1mM EDTA, 2M NaCl), and added to RNA/oligo annealing solution. After head-over-tail rotation at room temperature for 1 hr, beads were washed five times with 1X B&W buffer, bound viral RNA was eluted with 100mM DTT, and cleaned on Qiagen RNeasy mini columns using the manufacturer's protocol. Viral RNA enrichment was confirmed by RT-qPCR. Nucleosides were generated from 200ng purified RNA as previously described (Dominissini et al., 2016). Briefly, digestion was performed with nuclease P1 (Sigma, 2U) in buffer containing 25mM NaCl and 2.5mM ZnCl2 for 2h at $37^{0}$C, followed by incubation with Antarctic Phosphatase (NEB, 5U) for an additional 2h at $37^{0}$C. Nucleosides were separated and quantified using UPLC-MS/MS as previously described [Basanta-Sanchez et al.], except acetic acid was used in place of formic acid (Basanta-Sanchez et al., 2016).

## SUPPLEMENTAL REFERENCES


Aligeti, M., Roder, A., and Horner, S.M. (2015). Cooperation between the Hepatitis C Virus p7 and NS5B Proteins Enhances Virion Infectivity. J Virol *89*, 11523-11533.

Basanta-Sanchez, M., Temple, S., Ansari, S.A., D'Amico, A., and Agris, P.F. (2016). Attomole quantification and global profile of RNA modifications: Epitranscriptome of human neural stem cells. Nucleic Acids Res *44*, e26.

Corcoran, D.L., Georgiev, S., Mukherjee, N., Gottwein, E., Skalsky, R.L., Keene, J.D., and Ohler, U. (2011). PARalyzer: definition of RNA binding sites from PAR-CLIP short-read sequence data. Genome Biol *12*, R79.

Dominissini, D., Nachtergaele, S., Moshitch-Moshkovitz, S., Peer, E., Kol, N., Ben-Haim, M.S., Dai, Q., Di Segni, A., Salmon-Divon, M., Clark, W.C.*, et al.* (2016). The dynamic N(1)-methyladenosine methylome in eukaryotic messenger RNA. Nature *530*, 441-446.

Hafner, M., Landthaler, M., Burger, L., Khorshid, M., Hausser, J., Berninger, P., Rothballer, A., Ascano, M., Jr., Jungkamp, A.C., Munschauer, M.*, et al.* (2010). Transcriptome-wide identification of RNA-binding protein and microRNA target sites by PAR-CLIP. Cell *141*, 129-141.

Kennedy, E.M., Bogerd, H.P., Kornepati, A.V., Kang, D., Ghoshal, D., Marshall, J.B., Poling, B.C., Tsai, K., Gokhale, N.S., Horner, S.M.*, et al.* (2016). Posttranscriptional m(6)A Editing of HIV-1 mRNAs Enhances Viral Gene Expression. Cell Host Microbe *19*, 675-685.

Langmead, B., Trapnell, C., Pop, M., and Salzberg, S.L. (2009). Ultrafast and memory-efficient alignment of short DNA sequences to the human genome. Genome Biol *10*, R25.

Mali, P., Yang, L., Esvelt, K.M., Aach, J., Guell, M., DiCarlo, J.E., Norville, J.E., and Church, G.M. (2013). RNA-guided human genome engineering via Cas9. Science *339*, 823-826.

Meuleman, P., Catanese, M.T., Verhoye, L., Desombere, I., Farhoudi, A., Jones, C.T., Sheahan, T., Grzyb, K., Cortese, R., Rice, C.M.*, et al.* (2012). A human monoclonal antibody targeting scavenger receptor class B type I precludes hepatitis C virus infection and viral spread in vitro and in vivo. Hepatology *55*, 364-372.

Saito, T., Hirai, R., Loo, Y.M., Owen, D., Johnson, C.L., Sinha, S.C., Akira, S., Fujita, T., and Gale, M., Jr. (2007). Regulation of innate antiviral defenses through a shared repressor domain in RIG-I and LGP2. Proc Natl Acad Sci U S A *104*, 582-587.

Schindelin, J., Arganda-Carreras, I., Frise, E., Kaynig, V., Longair, M., Pietzsch, T., Preibisch, S., Rueden, C., Saalfeld, S., Schmid, B.*, et al.* (2012). Fiji: an open-source platform for biological-image analysis. Nat Methods *9*, 676-682.

Yamane, D., McGivern, D.R., Wauthier, E., Yi, M., Madden, V.J., Welsch, C., Antes, I., Wen, Y., Chugh, P.E., McGee, C.E.*, et al.* (2014). Regulation of the hepatitis C virus RNA replicase by endogenous lipid peroxidation. Nat Med *20*, 927-935.
